# Identification of *Mycobacterium tuberculosis* intracellular survival-related virulence factors via CRISPR-based eukaryotic-like secretory protein mutant library screen

**DOI:** 10.1128/spectrum.00767-25

**Published:** 2025-06-12

**Authors:** Weiyi Liu, Yingchao Wang, Jiayuan Zhao, Xinyue Zhang, Zihui Li, Hongyan Jia, Chuanzhi Zhu, Lanyue Zhang, Liping Pan, Zongde Zhang

**Affiliations:** 1 Beijing Key Laboratory for Drug Resistance Tuberculosis Research, Beijing Chest Hospital, Capital Medical University, Beijing Tuberculosis and Thoracic Tumor Research Institute 117550, Beijing, China; Shenzhen University School of Medicine, Shenzhen, China

**Keywords:** *Mycobacterium tuberculosis*, macrophage, CRISPR screen, eukaryotic-like secretory protein, virulence effector

## Abstract

Tuberculosis (TB), caused by *Mycobacterium tuberculosis* (*M.tb*), remains a serious infectious disease posing significant global health challenges. A critical evolutionary feature of *M.tb* is its genome encoding a set of eukaryotic-like secretory proteins, which facilitate intracellular survival by manipulating host immune responses. However, the specific eukaryotic-like secretory proteins that facilitate *M.tb* intracellular survival and their regulatory mechanisms on host immunity remain uncharacterized. In this study, a mutant library comprising 137 potential eukaryotic-like secretory proteins was constructed using clustered regularly interspaced short palindromic repeats (CRISPR)-non-homologous end joining genome editing technology. Subsequently, macrophages were infected with the mutant library, and CRISPR sequencing enabled preliminary identification of virulence factors associated with bacterial intracellular persistence. To validate the screen, two genes (*Rv0066c* and *Rv3139*) exhibiting the most pronounced reduction in intracellular survival rates when mutated were selected for the construction of large-fragment knockout strains (Δ*Rv0066c* and Δ*Rv3139*). Subsequent macrophage infection assays reconfirmed the impaired intracellular survival of these two mutants. RNA-seq analysis was conducted to characterize host gene expression profiles during Δ*Rv0066c*-infected macrophage interactions. RNA-seq analysis of macrophages infected with wild-type and Δ*Rv0066c* strains identified 138 differentially expressed genes, with 75 upregulated and 63 downregulated in Δ*Rv0066c*. Gene ontology clustering of these differentially expressed genes highlighted molecular functions related to chemokine binding, chemokine-mediated signaling pathways, Ras protein signal transduction, and calcineurin-mediated signaling. Collectively, this work established a potential eukaryotic-like secretory protein mutant library and identified two novel *M.tb* effectors governing intracellular survival, providing potential new targets for anti-TB drug development.

**IMPORTANCE:**

Eukaryotic-like secretory proteins that subvert host immunity to enable intracellular persistence are a key evolutionary adaptation of *Mycobacterium tuberculosis* (*M.tb*). In this study, we established a mutant library targeting 137 potential eukaryotic-like secretory proteins through clustered regularly interspaced short palindromic repeats (CRISPR)-non-homologous end joining genome editing technology. The library was subjected to macrophage infection assays, and CRISPR sequencing enabled identification of *M.tb* persistence-associated virulence determinants. Validation screens highlighted two genes (*Rv0066c* and *Rv3139*) that displayed the most significant intracellular survival defects to generate large-fragment knockout strains (Δ*Rv0066c* and Δ*Rv3139*). Macrophage infection experiments reconfirmed the compromised intracellular viability of both mutants. RNA-seq profiling of Δ*Rv0066c*-infected macrophages identified 138 differentially expressed genes, with functional enrichment in chemokine signaling, Ras protein signal transduction, and calcineurin-mediated signaling. To conclude, this study identified two novel *M.tb* effectors contributing to intracellular survival as potential new targets for anti-TB drug development.

## INTRODUCTION

Tuberculosis (TB), caused by the intracellular pathogen *Mycobacterium tuberculosis* (*M.tb*), is a highly infectious airborne disease (1), which claims millions of lives worldwide annually and remains a critical global public health challenge. Macrophages serve as the primary defense of the host immune system against *M.tb* and play a crucial role in determining the progression of TB (2, 3). A notable feature of *M.tb* evolution is its possession of a set of eukaryotic-like secretory proteins (4), which manipulate host immune mechanisms through diverse strategies (5, 6). These eukaryotic-like secretory proteins have become major therapeutic targets for anti-TB drugs.

Within the genome of *M.tb* H37Rv, 540 genes encode proteins with eukaryotic-like motifs or domains, among which 201 proteins are classified as *M.tb* potential secretory proteins (4, 7). Previous studies have demonstrated that Rv1988, a functional methyltransferase secreted by *M.tb*, can localize to the host cell nucleus and directly modify host chromatin. This protein catalyzes methylation at the H3R42 site of histone H3, thereby suppressing the expression of genes associated with anti-mycobacterial immunity. *M.tb* lacking Rv1988 exhibits significantly impaired survival capacity in host organisms (8). Additionally, Wang et al. revealed that the secreted tyrosine protein tyrosine phosphatase A (PtpA) promotes intracellular survival of *M.tb* by competitively binding to the RING domain of host TRIM27 (tripartite motif containing 27) to diminish its pro-apoptotic function, while concurrently targeting the JNK/p38 MAPK signaling cascades (9). Previous work by Kim et al. demonstrated that *M.tb* enhanced intracellular survival protein promotes *M.tb* survival in macrophages by acetylating dual-specificity protein phosphatase 16/mitogen-activated protein kinase phosphatase-7, thereby inhibiting JNK-dependent autophagy, phagosome maturation, and reactive oxygen species (ROS) production, ultimately suppressing host immune responses (10). However, the functions of many other eukaryotic-like secretory proteins remain unknown. Therefore, identifying virulence factors among *M.tb*-secreted eukaryotic-like proteins and elucidating their roles in TB pathogenesis will provide critical insights for developing improved therapeutic strategies.

To systematically investigate critical virulence factors among these potential eukaryotic-like secretory proteins, we established a mutant library of 137 genes using the clustered regularly interspaced short palindromic repeats (CRISPR)-Cas9-mediated genome editing coupled with non-homologous end joining (NHEJ)-driven repair mechanism (11). The library was screened via macrophage infection models, and CRISPR sequencing enabled the identification of virulence genes associated with *M.tb* intracellular survival. Genes exhibiting the most pronounced reduction in intracellular survival rates were selected for the construction of large-fragment knockout strains to validate the screens, followed by a preliminary exploration of how the encoded proteins promote *M.tb* persistence. This study further demonstrated that the application of CRISPR-assisted genome editing to construct a mutant library and the use of macrophages for *in vitro* screening are effective methods to identify *M.tb* virulence genes. Furthermore, the newly identified virulence-associated proteins provide potential targets for anti-TB drug development.

## RESULTS

### Optimized single-guide RNA (sgRNA) selection for CRISPR-mediated knockout of genes encoding eukaryotic-like secretory proteins in *M.tb*

**Fig 1:**
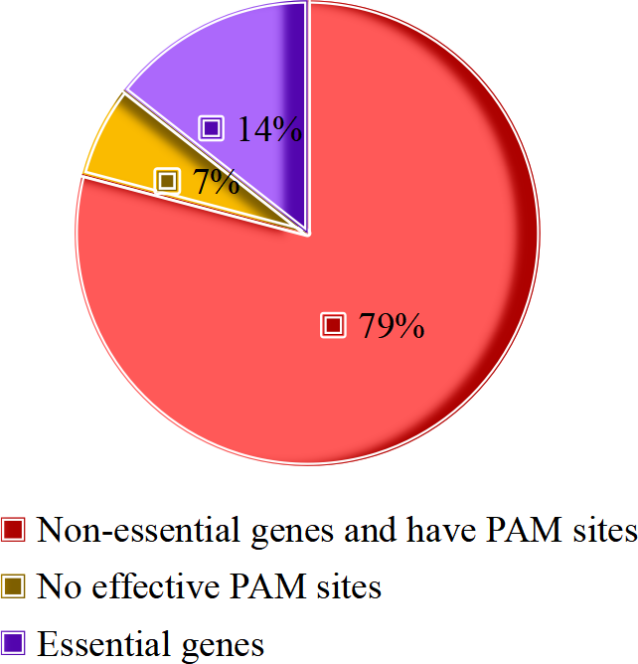
Classification of the 201 potential eukaryotic-like secretory proteins of *M.tb*. The 201 genes encoding potential eukaryotic-like secretory proteins in *M.tb were* classified as essential genes, non-essential genes, and those with or without effective PAM sites.

To construct a CRISPR-based mutant library of eukaryotic-like secretory proteins in *M.tb*, we first analyzed previously reported candidate genes encoding potential eukaryotic-like secretory proteins. By integrating annotations from the Mycobrowser database (https://mycobrowser.epfl.ch) and reviewing the literature, a total of 201 potential eukaryotic-like secretory proteins were classified. Among the 201 potential eukaryotic-like secretory proteins, 29 essential genes were deemed as necessary for *M.tb* survival as reported in a previous study (12). As the CRISPR-mediated genome editing approach requires protospacer adjacent motif (PAM) sites, our analysis of 172 non-essential genes revealed that 159 contained effective PAM sites while 13 lacked suitable PAM sites (Fig. 1). Therefore, after excluding the 42 genes, a total of 159 sgRNA sequences were designed for the construction of mutant strains (Table S1).

### Construction of the library of potential eukaryotic-like secretory protein mutants

**Fig 2:**
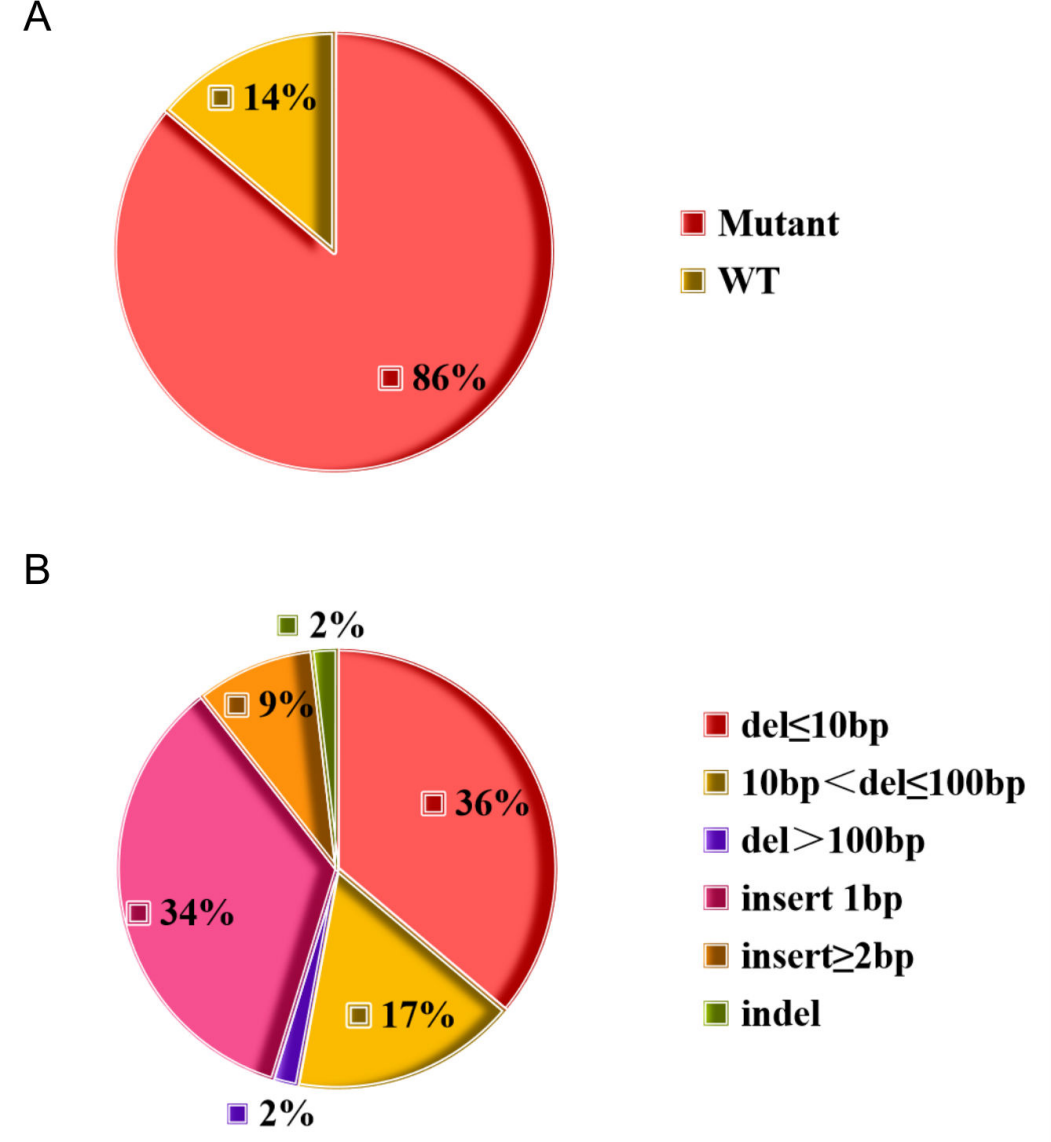
Statistics of the number and mutant information of *M.tb* potential eukaryotic-like secretory protein mutant strains. (**A**) Genome editing efficiency of the *M.tb* potential eukaryotic-like secretory protein mutant library. (**B**) Frequency of deletions or insertions detected in the mutant strains.

To investigate the roles of these potential eukaryotic-like secretory proteins in maintaining *M.tb* intracellular survival, CRISPR-assisted genomic editing technology (11) was used to construct a library of potential eukaryotic-like secretory protein mutant strains in *M.tb* H_37_Rv strain. During the process of building the mutant library, knockout mutants of 22 genes could not be produced. Eventually, a library of 137 potential eukaryotic-like secretory protein mutants was successfully constructed, covering 86.2% (137/159) of the target genes (Fig. 2A), with two independent mutants generated for each gene (total of 274 mutants). The mutation spectrum analysis was as follows: small fragment deletions (1 bp–10 bp): 36%; large-fragment deletions (10 bp–100 bp): 17%; extra large-fragment deletions (>100 bp): 2%; insertion mutations: 45% (Fig. 2B). All mutants were confirmed by colony PCR and sequencing, with mutation sites documented. Individual mutant strains were cultured and adjusted to an OD_600_ of 0.4 before cryopreservation at −80°C. The 274 mutants of 137 genes and three control strains (empty vector, non-targeting sgRNA) were mixed in a 1:1 ratio to form a functional screening library of *M.tb* potential eukaryotic-like secretory protein mutants for subsequent human monocyte leukemia cell line (THP-1)-derived macrophage infection assays.

### Intracellular survival analysis of eukaryotic-like secretory protein mutants using macrophage infection models

**Fig 3:**
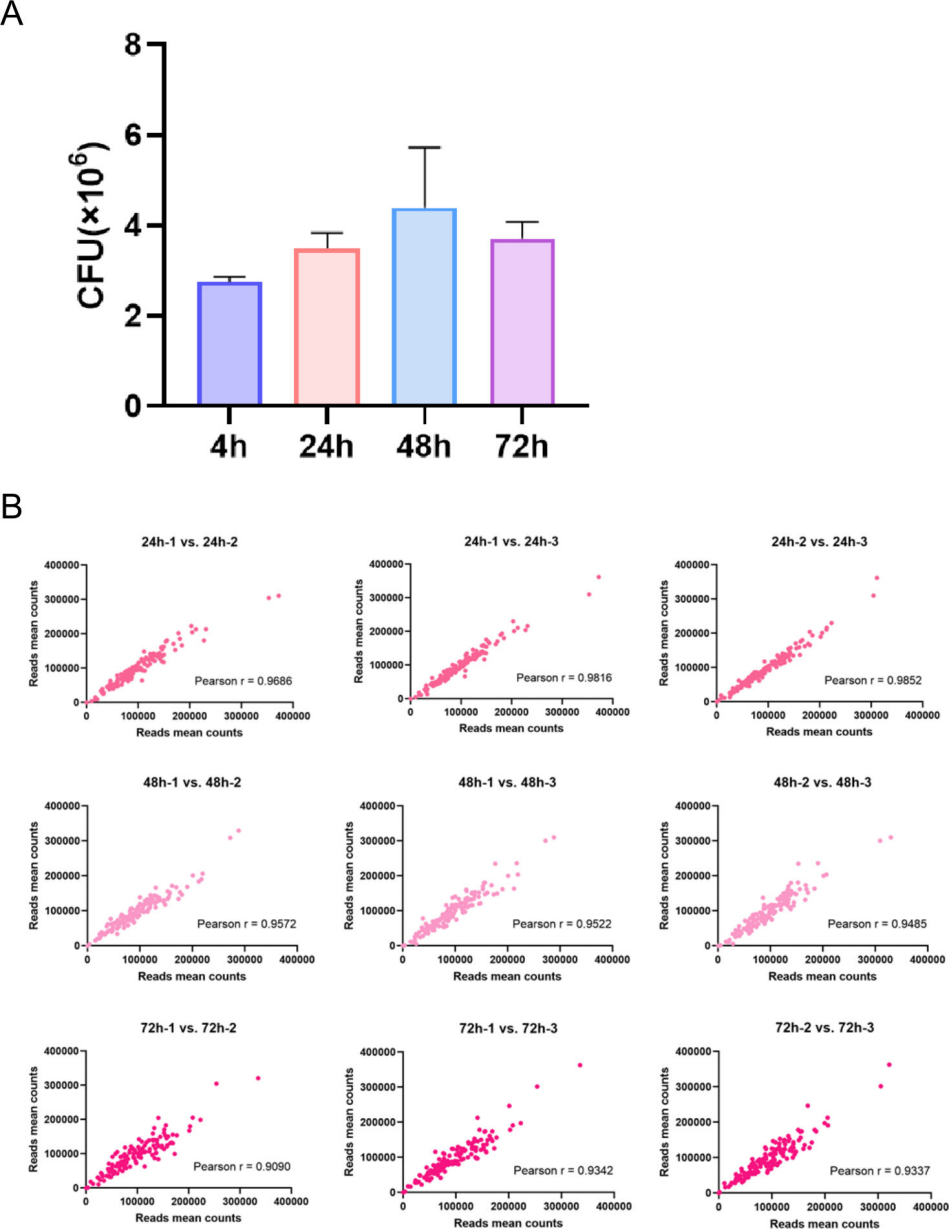
Analysis of the potential eukaryotic-like secretory protein mutant library colonies. (**A**) Statistics of the bacterial loads after THP-1 macrophage infection with the *M.tb* potential eukaryotic-like secretory protein mutant library at 4 h, 24 h, 48 h, and 72 h post-infection. (**B**) Pearson’s correlation coefficient analysis of the potential eukaryotic-like secretory protein mutant library.

To explore the role of these potential eukaryotic-like secretory proteins toward *M.tb* pathogenicity, THP-1-derived macrophages were infected with the mutant library. Genes that affected the intracellular survival of *M.tb* were identified by analyzing the fitness of different mutants within the infected macrophages. At 4, 24, 48, and 72 h post-infection, THP-1-derived macrophages were lysed, serially diluted, and plated on Middlebrook 7H10 agar for colony enumeration. As shown in Fig. 3A, 4 h after infection, each dish was colonized by approximately 2.5 × 10⁶ bacteria, indicating adequate coverage. At 24, 48, and 72 h post-infection, each dish was colonized by approximately 3.4 × 10⁶, 4.4 × 10⁶, and 3.7 × 10⁶ bacteria, respectively. The analysis of *M.tb* colony-forming unit (CFU) counts within the infected macrophages at different time points revealed that the *M.tb* library of potential eukaryotic-like secretory protein mutants progressively proliferated inside the macrophages, indicating the successful establishment of a cell-based screening model. The correlation analysis of reads from each replicate library showed that all Pearson’s correlation coefficients were above 0.9, indicating high reproducibility and good correlation of the screening results, which were suitable for subsequent screening and analysis (Fig. 3B).

**Fig 4:**
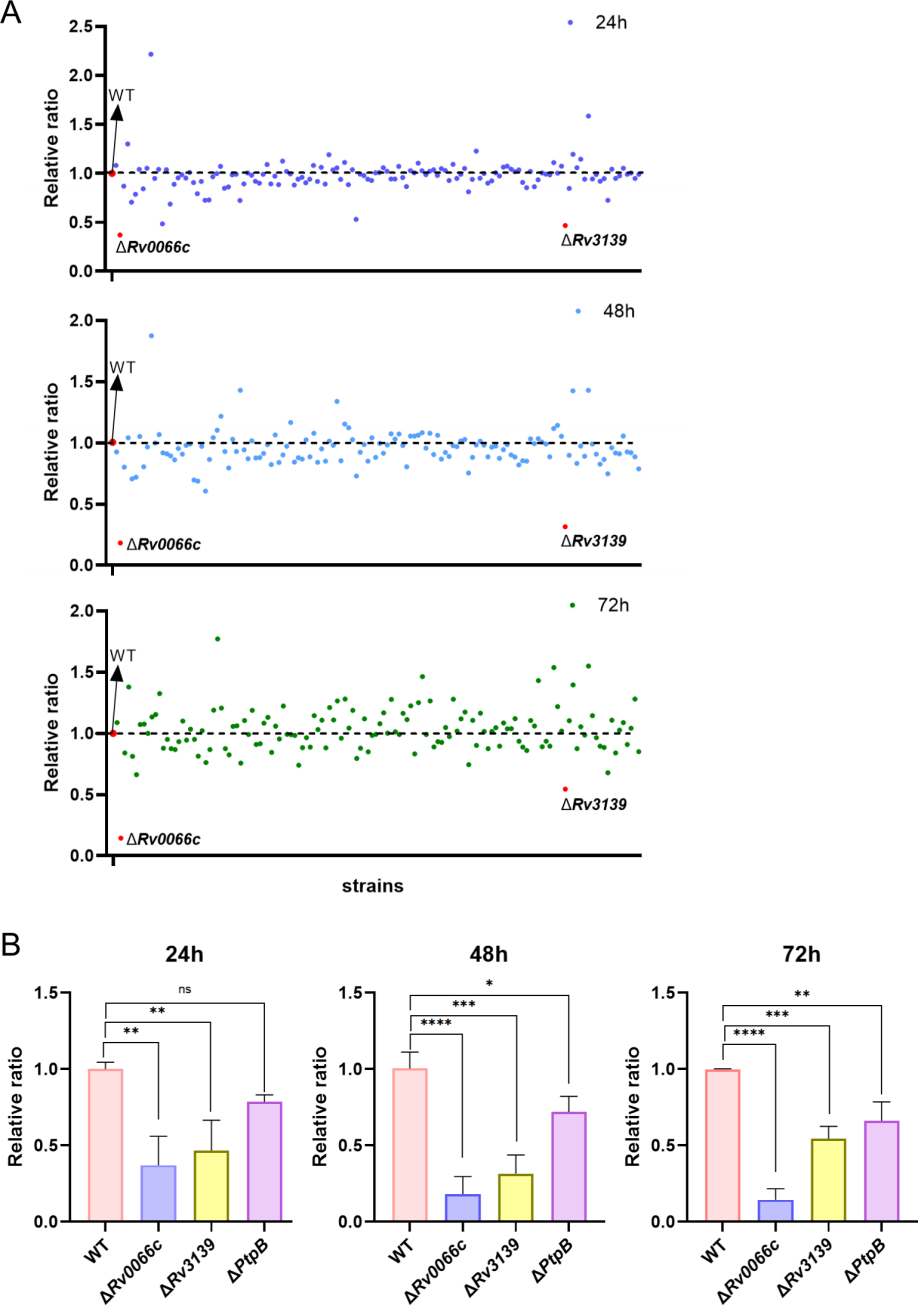
CRISPR sequencing identifies mutants with compromised survival in THP-1 cells. (**A**) The *M.tb* potential eukaryotic-like secretory protein mutant library was used to infect THP-1 macrophages. At 24 h, 48 h, and 72 h post-infection, cells were harvested and genomic DNA extracted. CRISPR sequencing was used to analyze the abundance of sgRNA in different knockout strains. (**B**) Relative ratio of Δ*Rv0066c*, Δ*Rv3139,* and Δ*PtpB* (Δ*Rv0153c*) strains. Data are presented as the mean ± SD of three independent experiments. Error bars are mean ± SD of three groups. Statistical significance was determined using one-way analysis of variance (Dunnett’s multiple comparisons test). n.s., not significant. *P < 0.05, **P < 0.01, ***P < 0.001, ****P < 0.0001.

### Screening for genes affecting *M.tb* intracellular survival in macrophages by CRISPR sequencing

At 4 h, 24 h, 48 h, and 72 h post-infection of THP-1 macrophages with the *M.tb* potential eukaryotic-like secretory protein mutant library, 2 × 10⁶ to 4 × 10⁶ CFUs were harvested from each of three cell culture dishes at every time point. Genomic DNA was extracted, and the sgRNA regions were PCR-amplified for CRISPR sequencing and analysis (Fig. 4A). Quantifying the abundance of sgRNA for each mutant allowed us to assess the replication and expansion frequency of *M.tb* within THP-1 macrophages. Based on the sequencing results, mutants of *Rv0066c* and *Rv3139*, whose sgRNA reads dropped significantly at 24 h, 48 h, and 72 h post-infection compared to the wild-type (WT) control strain, were identified. When analyzing the screening results, the *Rv0153c* (PtpB) mutant within the library was used as a positive control to confirm the accuracy of our library screen (Fig. 4B).

### *In vitro* growth kinetics assay of potential virulence factors

**Fig 5:**
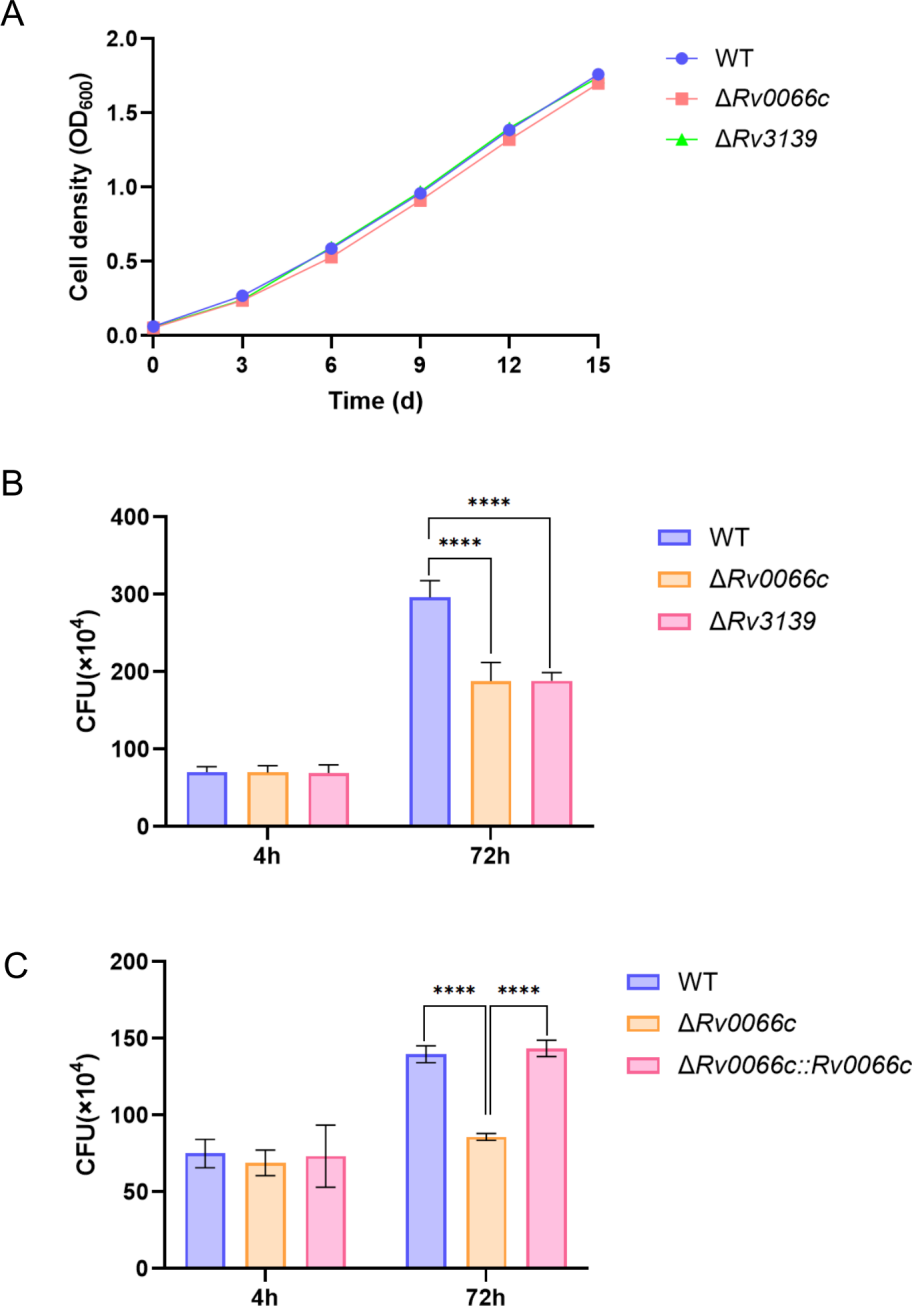
Rv0066c and Rv3139 promote *M.tb* survival in macrophages. (**A**) *In vitro* growth kinetics of the WT, Δ*Rv0066c*, and Δ*Rv3139* strains in Middlebrook 7H9 broth media (*n* = 3). (**B**) THP-1 cells were infected with WT, Δ*Rv0066c*, or Δ*Rv3139* strains at a multiplicity of infection (MOI) of 10 for 4 h and 72 h. Bacterial loads in cells at the indicated time points after infection were determined and represented as CFU. (**C**) THP-1 cells were infected with WT, Δ*Rv0066c*, or Δ*Rv0066c::Rv0066c* strains at an MOI of 10 for 4 h and 72 h. Bacterial loads in cells at the indicated time points after infection were determined as CFU. Data are presented as the mean ± SD of three independent experiments. Error bars are mean ± SD of three groups. Statistical significance was determined using one-way analysis of variance (Dunnett’s multiple comparisons test). ****P < 0.0001.

To further investigate whether proteins encoded by *Rv0066c* and *Rv3139* promote *M.tb* survival during infection, we constructed large-fragment knockout strains of *Rv0066c* (Δ*Rv0066c*) and *Rv3139* (Δ*Rv3139*) using CRISPR-assisted genome editing. Sequencing analysis confirmed successful deletion of 2,017 bp and 1,193 bp fragments encoding Rv0066c and Rv3139, respectively, in the *M.tb* genome (Fig. S1). Stable genetic traits and growth characteristics are conducive to guiding the analysis of cell infection experiments. As gene knockouts in *M.tb* may affect bacterial growth, we measured the *in vitro* growth kinetics of the WT strain, Δ*Rv0066c* strain, and Δ*Rv3139* strain in 7H9 liquid complete medium. When compared to the WT control strain, the growth rates of the Δ*Rv0066c* and Δ*Rv3139* strains in the medium were not significantly different, and the growth curves were similar (Fig. 5A). These results indicate that the Δ*Rv0066c* and Δ*Rv3139* strains did not affect extracellular replication of *M.tb* or bacterial growth *in vitro*.

### Confirmation that Rv0066c and Rv3139 promote *M.tb* survival in macrophages

To confirm that proteins encoded by the two genes *Rv0066c* and *Rv3139*, identified by sequencing, affect intracellular bacterial survival, THP-1 macrophages were infected with the WT, Δ*Rv0066*c, or Δ*Rv3139* strains at a multiplicity of infection (MOI) of 10 for 4 h and 72 h, and intracellular bacterial loads were measured. There were no significant differences in bacterial load among the three groups at 4 h, indicating equivalent phagocytosis of the three strains by THP-1 macrophages. However, at 72 h post-infection, the intracellular bacterial load of Δ*Rv0066c* and Δ*Rv3139* strains in THP-1 macrophages was significantly lower than that of the WT strain (*P* < 0.0001). This suggests that secretory proteins encoded by *Rv0066c* and *Rv3139* promote *M.tb* survival in THP-1 macrophages and that knocking out these genes significantly inhibits intracellular survival (Fig. 5B). Furthermore, the Δ*Rv0066c* mutant was complemented and used to infect macrophages for functional validation of its role in *M.tb* survival. The complemented strain Δ*Rv0066c::Rv0066c* exhibited restored intracellular survival capacity in macrophages compared to the deletion mutant Δ*Rv0066c* (Fig. 5C).

### Transcriptome profiling reveals macrophage gene expression modulation by Δ*Rv0066c* strain infection

**Fig 6:**
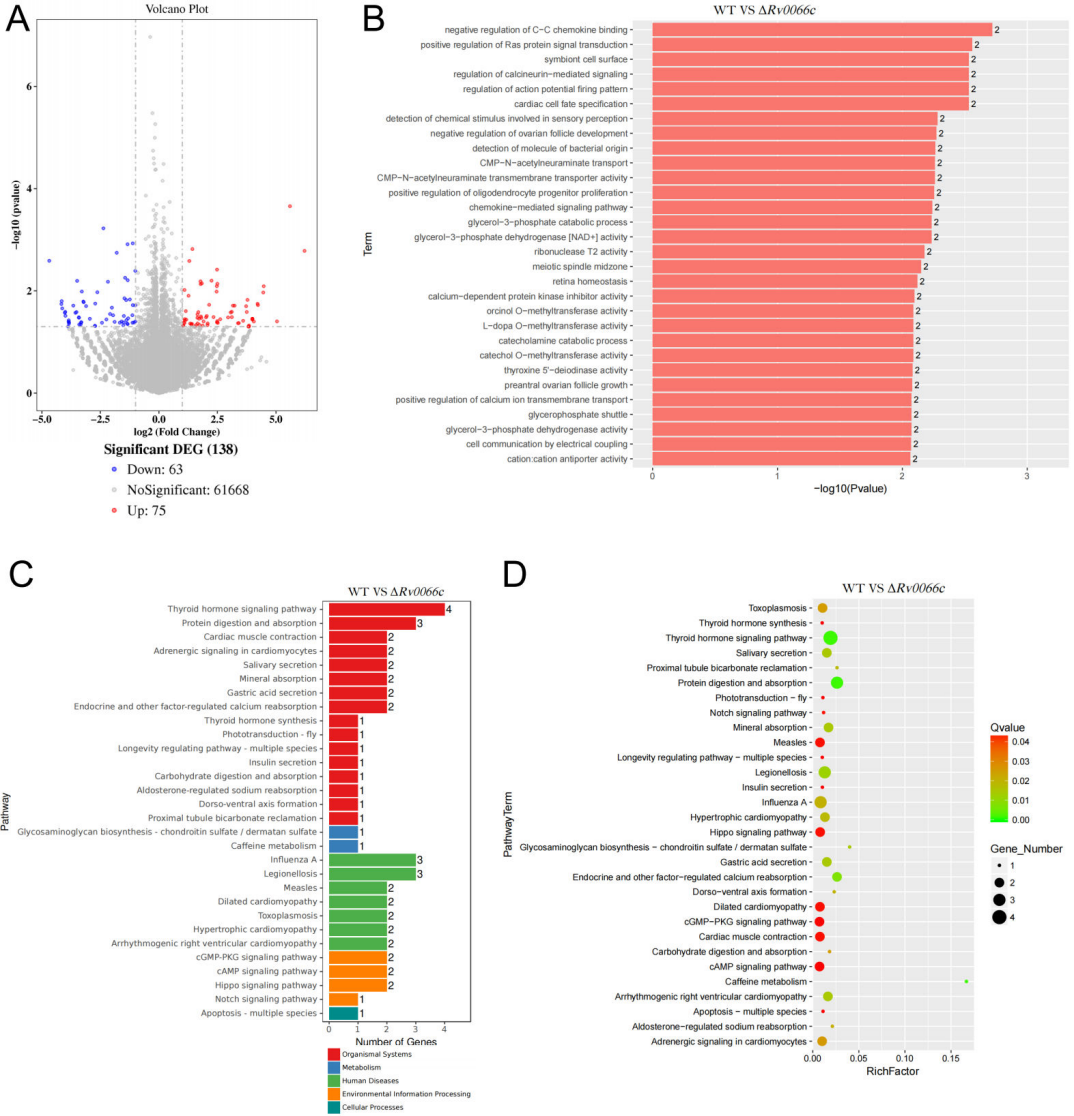
Transcriptome profiling reveals modulation of macrophage gene expression following Δ*Rv0066c* infection. (**A**) The volcano plot illustrates the expression profiles of differentially expressed genes in THP-1 macrophages infected with the WT strain versus the Δ*Rv0066c* strain. The *y*-axis and *x*-axis represent the *P*-value and fold change in expression for each gene, respectively. Statistically significant upregulated and downregulated genes are highlighted in red and blue, respectively. (**B**) Bar plot of *P*-values for molecular function GO enrichment analysis of differentially expressed genes. The vertical axis displays enriched GO terms, while the horizontal axis represents the corresponding log10 (*P*-value) values. (**C**) Bar chart of significantly enriched KEGG annotation categories. The *y*-axis shows pathway names, and the *x*-axis shows the number of genes. (**D**) KEGG enrichment scatter plot of differentially expressed genes. The *y*-axis shows pathway names, and the x-axis shows the Rich factor. The size of the dots indicates the number of differentially expressed genes in the pathway, and the color corresponds to different Q-value ranges. These data represent three biological replicates.

To elucidate how Rv0066c promotes intracellular survival of *M.tb*, phorbol-12-myristate-13-acetate (PMA)-differentiated THP-1 macrophages were infected with either WT or Δ*Rv0066c* strains at an MOI of 10 for 24 h, followed by RNA-sequencing (GENEWIZ, Inc.). Comparative transcriptome analysis between WT-infected and Δ*Rv0066c*-infected groups revealed 138 differentially expressed genes, with 75 upregulated and 63 downregulated genes in Δ*Rv0066c*-infected macrophages (Fig. 6A). The genes with significant differential expression (Top 10, |log2FC| > 1, *P*-value <0.05) and their functional annotations are shown in Table S2. Gene ontology clustering of these differentially expressed genes highlighted molecular functions related to chemokine binding, chemokine-mediated signaling pathways, Ras protein signal transduction, and calcineurin-mediated signaling (Fig. 6B). The Kyoto Encyclopedia of Genes and Genomes (KEGG) database, a comprehensive resource for functional annotation and integration of genomic data with higher-order biological networks (13), was referred to for pathway enrichment analysis. The top 30 enriched pathways identified through KEGG analysis were associated with thyroid hormone signaling, cyclic adenosine monophosphate (cAMP) signaling, apoptosis signaling, and others (Fig. 6C and D).

## DISCUSSION

During the interaction between the host immune system and pathogenic microorganisms, pathogens secrete complex molecules known as effectors to subvert host immune responses. As a highly successful intracellular pathogen, *M.tb* has co-evolved extensively with human hosts. It has evolved various effector proteins to escape host innate and adaptive immunity, ensuring its intracellular survival and driving TB development and progression. A notable evolutionary feature of *M.tb* is its eukaryotic-like secretory proteins, acquired through horizontal gene transfer and convergent evolution (4). These proteins play crucial roles in regulating host immune responses and facilitating *M.tb* intracellular survival (4, 14). Understanding the functional roles of these secretory proteins in TB pathogenesis and developing therapeutic approaches targeting these proteins are of paramount importance (15). Guided by previous reports on 201 potential eukaryotic-like secretory proteins in *M.tb* (4, 7), we employed the CRISPR-assisted genome editing technology and established a mutant library encompassing 137 potential eukaryotic-like secretory proteins. Subsequently, through CRISPR sequencing, two novel virulence factors, Rv0066c and Rv3139, were screened and validated as influencing *M.tb* intracellular survival.

Prior to constructing the mutant library, we found that among the 201 potential eukaryotic-like secretory proteins, there were 29 essential genes including *Rv0014c* (*PknB*), *Rv0015c* (*PknA*), *Rv0018c* (*PstP*), *Rv0046c* (*Ino1*), *Rv0285* (*PE5*), *Rv0291* (*MycP3*), *Rv0558* (*MenH*), *Rv1093* (*GlyA1*), *Rv1423* (*WhiA*), and *Rv3303c* (*LpdA*). These genes were previously verified as necessary for bacterial survival and mainly involved in core pathways such as cell wall synthesis and energy metabolism (12, 16). Among the 172 non-essential genes of potential eukaryotic-like secretory proteins, 13 genes without effective PAM sites were excluded. Five of these genes are PE/PPE family genes *Rv0978c* (*PE_PGRS17*), *Rv1790* (*PPE27*), *Rv2353c* (*PPE39*), *Rv2356c* (*PPE40*), and *Rv3872* (*PE35*). Their high sequence repetition prevents designing unique knockout sites. However, PE/PPE family genes have previously been reported to be related to virulence (17, 18), so the omission of these genes from this study has limited consequences on the discovery of new virulence factors. Eight genes that lack PAM sites include two related to intermediate metabolism and respiration, *Rv3117* (*CysA3*) (19) and *Rv3251c* (*rubA*) (20), as well as three linked to cell wall and cellular processes, which are *Rv1081c*, *Rv1615*, and *Rv3615c* (*EspC*) (21). There were only three genes with unknown functions: *Rv0336*, *Rv0546c*, and *Rv3686c*. During the process of constructing the mutant library, knockout mutants of 22 genes, including *Rv0410c* (*PknG*), *Rv0442c* (*PPE10*), *Rv0733* (*Adk*), and *Rv3159c* (*PPE53*), could not be generated, likely due to inefficient sgRNA design. We successfully constructed a mutant library of 137 potential eukaryotic-like secretory proteins, covering 86.2% of the target proteins. Through CRISPR sequencing and analysis, we identified that *Rv0066c* and *Rv3139* mutants exhibited significantly reduced survival in macrophages. A significant reduction in the survival of the *Rv0153c* mutant within macrophages was also noted. Protein tyrosine phosphatase B (PtpB, Rv0153c) is an *M.tb*-secreted tyrosine phosphatase (22–24). Studies have identified PtpB as a critical virulence factor in *M.tb,* where PtpB suppresses macrophage apoptosis by inhibiting p53 expression, thereby promoting *M.tb* survival (25). A separate study revealed its capacity to inhibit pyroptosis through modulation of host membrane composition, enabling *M.tb* evasion of host immune responses (4). These findings support the feasibility and reliability of our screening approach.

While previous bioinformatics-based studies have shown that *Rv0066c* and *Rv3139* encode *M.tb* secretory proteins (26, 27), their functional contribution to intracellular bacterial persistence had yet to be established. In this study, the construction of large-fragment deletion mutants (Δ*Rv0066c* and Δ*Rv3139*) combined with macrophage infection assays further validated their critical association with *M.tb* intracellular survival. Key enzymes such as isocitrate dehydrogenase (ICD), pyruvate kinase, and succinate dehydrogenase are reported to play a crucial role in the growth and energy metabolism of *M.tb* (28). *M.tb* Rv0066c (ICD-2) is an isocitrate dehydrogenase (29), and is reported to participate in the tricarboxylic acid (TCA) cycle by catalyzing the oxidative decarboxylation of isocitrate, providing energy and metabolic intermediates for the bacterium (30, 31). A recent report indicated that metabolic dysregulation in *M.tb* can significantly increase macrophage apoptosis or pyroptosis (32). Knocking out genes closely related to bacterial respiration and intermediate metabolism can abolish *M.tb*’s suppression of macrophage apoptosis or pyroptosis (32), reducing its virulence capacity. These findings highlight the importance of *M.tb*’s metabolic plasticity in its pathogenicity within the host.

In our study, comparative transcriptome analysis demonstrated that Rv0066 may be functionally linked to macrophage apoptotic pathways. Host immune system-induced apoptosis regulates intracellular bacterial viability (33), restricts pathogen dissemination by preventing microbial release (34), and enhances host control of bacterial infections (35), playing a pivotal role in antimicrobial defense. For instance, PtpA, a low-molecular-weight secretory tyrosine phosphatase essential for *M.tb* intracellular survival (9), interacts with and dephosphorylates host glycogen synthase kinase 3α, thereby suppressing apoptosis and improving bacterial survival within host cells (36). These findings suggest that Rv0066c may regulate apoptosis through similar or novel mechanisms. Rv3139, a probable acyl-CoA dehydrogenase (FadE24), belongs to the FadE gene family, which is critical for cholesterol metabolism in *M.tb*, with *FadE* gene expression regulated by cholesterol. This suggests that the *FadE*-encoded protein is vital for *M.tb* adaptation to and utilization of host nutrients (37). Cholesterol is a key carbon source for *M.tb*’s survival and pathogenicity within the host, and *M.tb* can use host cell cholesterol as an energy source (38, 39). As ATP levels are closely related to *M.tb* growth and replication, host cholesterol could serve as an energy source for its activities (40). An activity-based protein analysis study suggested that FadE23 and FadE24 may bind ATP (41), indicating a possible catalytic function beyond acyl-CoA dehydrogenation.

This study has several limitations that warrant consideration. First, the macrophage infection model does not adequately mimic pathophysiological host niches. Second, the current investigation focused solely on complementation verification and RNA-sequencing analysis of the most statistically significant candidate, Rv0066c, leaving the mechanistic underpinnings of its virulence regulation unexplored and other candidates (e.g., Rv3139) unvalidated. Future work will systematically evaluate the mutant library in the *in vivo* murine infection models to identify genes critical for *M.tb* survival under physiologically relevant conditions and delineate molecular pathways by which Rv0066c/Rv3139 interact with host defenses.

In summary, this study employed a CRISPR-NHEJ genome editing approach to construct a library of *M.tb* potential eukaryotic-like secretory protein mutants. Adopting a strategy that focused on *M.tb*-host interactions, *in vitro* cellular screening identified critical genes promoting bacterial survival within host macrophages. These findings validate CRISPR-assisted genome editing combined with cell-based screens as an effective strategy to identify bacterial virulence genes, establishing a robust foundation for more extensive investigations on *M.tb* and other intracellular pathogens. The newly identified virulence genes provide novel therapeutic targets for advancing anti-TB drug development.

## MATERIALS AND METHODS

### Bacterial strain cultivation

The *Mycobacterium tuberculosis* H37Rv strain (ATCC: 27294) was inoculated into Middlebrook 7H9 medium (BD Biosciences) with 10% OADC (oleic acid, albumin, dextrose, and catalase) (BD Biosciences) and incubated at 37°C for 14 days or when the OD_600_ value was approximately 0.7, indicating the strain was in the logarithmic growth phase. The bacteria were then used for cell infection experiments. The bacterial strains utilized in this study are listed in Table S3.

### Cell culture

THP-1 cells were passaged and cultured in Roswell Park Memorial Institute (RPMI) 1640 medium (Gibco) supplemented with 10% fetal bovine serum (Gibco), at 37°C with 5% CO_2_ to the logarithmic growth phase. When the required cell numbers were achieved, the cells were plated, and PMA stock solution was added to a final concentration of 100 ng/mL. The THP-1 cells were incubated for ~36 h to differentiate them into macrophages, and PMA induction was stopped when more than 90% of the cells adhered to the plate. The cells were washed three times with serum-free RPMI 1640 medium and further incubated for about 15 h in complete medium without PMA.

### Bacterial CFU assay

Depending on the experimental goals, macrophages were infected with logarithmic-phase bacteria at an MOI of 10 and incubated at 37°C. After 4 h, the bacteria-containing medium was carefully replaced with RPMI 1640. Cells were washed three times to eliminate extracellular bacteria, after which fresh complete medium was added, and cells were further incubated. At each preset time point, cells were washed three times with RPMI 1640. After the media was removed, 500 µL of 0.5% Triton X-100 was added to each well. Plates were incubated at room temperature for 10 minutes, after which cells were disrupted by a pipetting action. Serial dilution of the cells was performed, and the bacteria were evenly spread onto 7H10 agar plates with 10% OADC and incubated at 37°C for 3–4 weeks to determine CFU at each dilution.

### Construction of the potential eukaryotic-like secretory protein mutant library

*M.tb* competent cells were prepared as previously described (11). The strain containing plasmids expressing NHEJ and RecX was cultured in roller bottles (Greiner Bio-one) with 7H9 medium at 37°C for 15 days. When the OD_600_ value reached 0.7, glycine stock solution was added to a final concentration of 1.5% and the roller bottles were incubated at 37°C for another 24 h. Bacteria were collected by centrifugation, washed three times with 10% glycerol, and finally resuspended in 10% glycerol.

One hundred thirty-seven sgRNA expression plasmids targeting potential eukaryotic-like secretory protein genes were constructed based on the vector pYC1446. Three sgRNAs that did not target any gene were used as controls. The prepared competent cells were mixed with the plasmids and transferred to an electroporation cuvette (Bio-Rad) for electroporation (11). After electroporation, bacteria were cultured in 7H9 medium for 24 h and spread onto 7H10 agar plates containing kanamycin (25 µg/mL), zeocin (50 µg/mL), and anhydrotetracycline (50 ng/mL). Transformants were picked for PCR and sequence analysis to confirm successful genome editing. Finally, the mutant library was formed by mixing 137 potential eukaryotic-like secretory protein mutants and three control strains at a ratio of 1:1. The plasmids utilized in this study are detailed in Table S4.

### Macrophage infection, CRISPR sequencing library preparation, and data analysis

THP-1 monocytes were seeded in 150 mm dishes and induced with PMA (100 ng/mL) for about 36 h, then cultured for another 15 h in PMA-free complete medium. Differentiated THP-1 macrophages were infected with the mutant library at an MOI of 10 and incubated at 37°C with 5% CO_2_. After 4 h, the medium with bacteria was removed, and cells were washed three times with RPMI 1640 to eliminate extracellular bacteria, then cultured in fresh complete medium. At 4 h, 24 h, 48 h, and 72 h post-infection, THP-1 cells were lysed, serially diluted, and spread onto antibiotic-free 7H10 agar plates, which were then incubated inverted at 37°C. Monoclonal colonies from each time point were scraped from the 7H10 plates with a spreader and resuspended in phosphate-buffered saline containing 0.05% Tween-80. Each sample collected contained at least 6 × 10³ CFU, and genomic DNA was extracted from the harvested bacteria. The sgRNA coding region was PCR-amplified using genomic DNA as the template. The PCR fragments were sent to GENEWIZ, Inc. (Suzhou, China) for amplicon sequencing. Then libraries with different indices were multiplexed and loaded on an Illumina Novaseq instrument. Sequencing was carried out using a 2 × 150 bp paired-end (PE) configuration (42). The sequencing results were analyzed using the MAGeCK (model-based analysis of genome-wide CRISPR-Cas9 knockout) robust ranking method (version 0.5.9.4) (43). During CRISPR sequencing, the read count was normalized relative to the total sgRNA reads in each sample.

### Construction of large-scale gene deletion knockout strains of *M.tb*

The Δ*Rv0066c* and Δ*Rv3139* mutants were constructed using the CRISPR-NHEJ gene editing strategy (11). Specifically, the respective sgRNA sequence was amplified with specific primers and ligated into the pYC1876 vector. The H37Rv strain was transduce and recombinants were screened on 7H10 agar plates containing kanamycin, zeocin, and ATc. Mutants were screened via PCR and confirmed by DNA sequencing. The pYC874-L5-Rv0066c plasmid (synthesized by GENEWIZ) was used for complementation of the Δ*Rv0066c* strain and defined as Δ*Rv0066c::Rv0066c*. The primer sequences used in this study are listed in Table S5.

### *In vitro* strain growth kinetics assay

To evaluate the impact of gene deletions on the *in vitro* growth of *M.tb*, the WT strain, Δ*Rv0066c* strain, and Δ*Rv3139* strain were inoculated into 7H9 liquid medium to measure *in vitro* growth kinetics. Each strain was inoculated at an OD_600_ of 0.04 and cultured to mid-log phase. Absorbance (OD_600_) was measured every 3 days for 15 days, and growth curves were plotted based on the recorded values.

### RNA-sequencing and analysis

The WT and Δ*Rv0066c* strains were used to infect differentiated THP-1 macrophages at an MOI of 10 for 24 h. Total RNA was extracted using the Total RNA Kit (OMEGA). Subsequently, RNA-seq analysis was performed on these samples by GENEWIZ, Inc. (Suzhou, China). One microgram of total RNA was used for the library preparation. Poly(A) mRNA isolation was performed using Oligo(dT) beads followed by mRNA fragmentation using divalent cations and high temperature. Priming was performed using random primers. First-strand cDNA and second-strand cDNA were synthesized. The purified double-stranded cDNA was then subjected to end repair and dA tailing in a single reaction, followed by T-A ligation to add adaptors at both ends. Size selection of adaptor-ligated cDNA was then performed using DNA Clean Beads. Each sample was then amplified by PCR using P5 and P7 primers, and the PCR products were validated. Then libraries with different indices were multiplexed and loaded on an Illumina Novaseq 6000 instrument for sequencing using a 2 × 150 bp PE configuration according to manufacturer’s instructions.

### Statistical analysis

Data are presented as mean ±  standard deviation of at least three biological replicates. One-way analysis of variance (Dunnett’s multiple comparisons test) was used for analysis of multiple groups with a single independent variable. The statistical analyses were performed using GraphPad Prism 8.0. *P* < 0.05 was defined as statistically significant.

## Data Availability

The data that support these findings are available from the corresponding author upon reasonable request. CRISPR Screen data and RNA-seq data in this study have been deposited at the NCBI Sequence Read Archive (SRA) with the accession number PRJNA1236249 (https://www.ncbi.nlm.nih.gov/sra/PRJNA1236249) and PRJNA1236621 (https://www.ncbi.nlm.nih.gov/sra/PRJNA1236621).

## SUPPLEMENTAL MATERIAL

Supplemental material spectrum.00767-25-s0001.docx Fig. S1; Tables S1 to S5.

ASM does not own the copyrights to Supplemental Material that may be linked to, or accessed through, an article. The authors have granted ASM a non-exclusive, world-wide license to publish the Supplemental Material files. Please contact the corresponding author directly for reuse.
