# Supplementary material for: Identification of Mycobacterium tuberculosis intracellular survival-related virulence factors via CRISPR-based eukaryotic-like secretory protein mutant library screen
Source: Microbiol Spectr. 2025 Jun 12;13(8):e00767-25. doi: 10.1128/spectrum.00767-25 (PMC12323669; doi:10.1128/spectrum.00767-25)
Supplement: Supplemental material — Fig. S1; Tables S1 to S5. [file spectrum.00767-25-s0001.docx]

**Supplementary Materials for Identification of** ***Mycobacterium tuberculosis* Intracellular Survival-Related Virulence Factors via CRISPR-Based Eukaryotic-Like Secretory Proteins Mutant Library Screen**


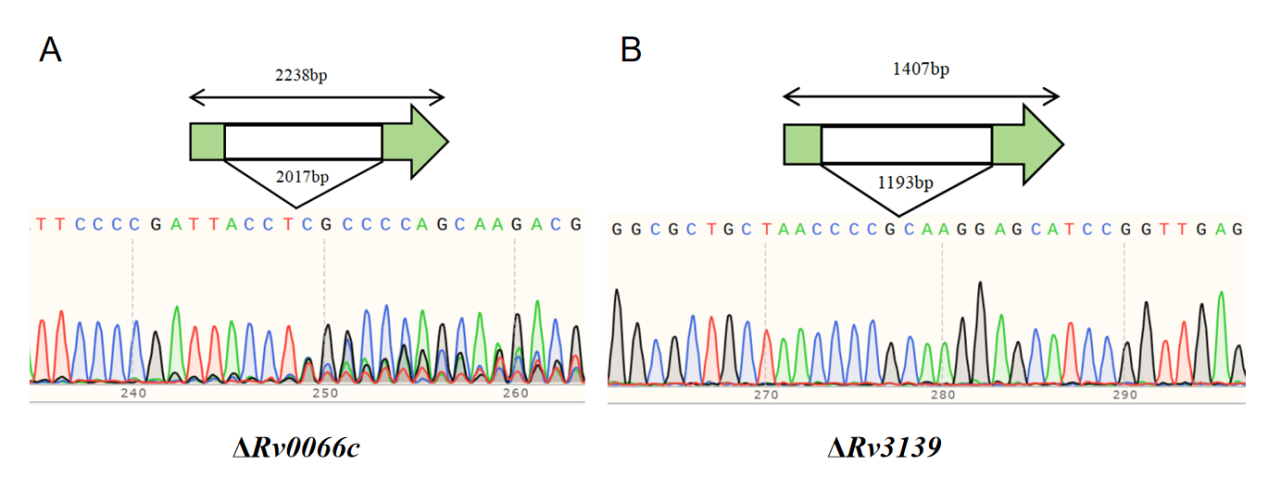


**Supplementary Figure 1. (A)** Δ***Rv0066c* and (B)** Δ***Rv3139* knockout strain validation sequencing plots.**

**Table S1. sgRNA sequence of the eukaryotic-like secreted protein in *M.tb***

| **number** | **gene** | **sgRNA-top (5’-3’)** | **sgRNA-bottom (5’-3’)** |
| --- | --- | --- | --- |
| 1 | Rv0062 | gggacgatgcggcaaacccgctgg | aaacccagcgggtttgccgcatcg |
| 2 | Rv0066c | gggagaattccccgattacctgac | aaacgtcaggtaatcggggaattc |
| 3 | Rv0089 | gggaaatgccgccctgcaccacat | aaacatgtggtgcagggcggcatt |
| 4 | Rv0116c | gggacgatcacgctgatgctcacc | aaacggtgagcatcagcgtgatcg |
| 5 | Rv0139 | gggactcgtcgccgactgcgcgcc | aaacggcgcgcagtcggcgacgag |
| 6 | Rv0153c | gggagtccgtgaactgccgggcgc | aaacgcgcccggcagttcacggac |
| 7 | Rv0169 | gggattggtatacctgcagtttcg | aaaccgaaactgcaggtataccaa |
| 8 | Rv0183 | gggagtgctggcccatggtctggg | aaaccccagaccatgggccagcac |
| 9 | Rv0187 | gggacgctagcgtttgggcatgga | aaactccatgcccaaacgctagcg |
| 10 | Rv0198c | gggaacctgatcatccaggccagc | aaacgctggcctggatgatcaggt |
| 11 | Rv0229c | gggaaaacatctcgtcgacatcga | aaactcgatgtcgacgagatgttt |
| 12 | Rv0260c | gggacttcccgacgacgatgaact | aaacagttcatcgtcgtcgggaag |
| 13 | Rv0274 | gggaggcgcgcaccgtggacttct | aaacagaagtccacggtgcgcgcc |
| 14 | Rv0305c | gggaatttcggcaacggcccaggc | aaacgcctgggccgttgccgaaat |
| 15 | Rv0386 | gggacatgacggcgtacgcccagt | aaacactgggcgtacgccgtcatg |
| 16 | Rv0410c | gggagacggctgggcggcggcctg | aaaccaggccgccgcccagccgtc |
| 17 | Rv0411c | gggactgccgccggacagttcgag | aaacctcgaactgtccggcggcag |
| 18 | Rv0425c | gggaaaccctggccaagaccgggg | aaacccccggtcttggccagggtt |
| 19 | Rv0426c | gggatggggcgaacggtgagtggt | aaacaccactcaccgttcgcccca |
| 20 | Rv0432 | gggatcgggactttcgggtcacga | aaactcgtgacccgaaagtcccga |
| 21 | Rv0435c | gggatacgtccacggccgggcaag | aaaccttgcccggccgtggacgta |
| 22 | Rv0442c | gggaccgcctggggcgagctggcc | aaacggccagctcgccccaggcgg |
| 23 | Rv0446c | gggacgcaagacagccggtcaggg | aaacccctgaccggctgtcttgcg |
| 24 | Rv0457c | gggaggctcgaggacgtcaccggt | aaacaccggtgacgtcctcgagcc |
| 25 | Rv0518 | gggaggcgaccacggcaacgtgtt | aaacaacacgttgccgtggtcgcc |
| 26 | Rv0561c | gggatgagcggcttcggaggcgag | aaacctcgcctccgaagccgctca |
| 27 | Rv0571c | gggattggctttcggcgccatcgg | aaacccgatggcgccgaaagccaa |
| 28 | Rv0577 | gggaccggaggggatgccgccgat | aaacatcggcggcatcccctccgg |
| 29 | Rv0594 | gggaacatatcggggcatcaccat | aaacatggtgatgccccgatatgt |
| 30 | Rv0618 | gggatgagcgccacgccaccgccc | aaacgggcggtggcgtggcgctca |
| 31 | Rv0622 | gggagaagccgttgacccacttgg | aaacccaagtgggtcaacggcttc |
| 32 | Rv0629c | gggaatctggaccgctactggcgc | aaacgcgccagtagcggtccagat |
| 33 | Rv0648 | gggacgcttcgtgtgcgcgctgcc | aaacggcagcgcgcacacgaagcg |
| 34 | Rv0654 | gggatgccggtcaccggccgcatt | aaacaatgcggccggtgaccggca |
| 35 | Rv0669c | gggatgcccatgcaaaacgtgaat | aaacattcacgttttgcatgggca |
| 36 | Rv0671 | gggatgcgttcgcgggctgctcgg | aaacccgagcagcccgcgaacgca |
| 37 | Rv0733 | gggaacgcaggcggtgaagctggc | aaacgccagcttcaccgcctgcgt |
| 38 | Rv0755c | gggaggggcctgggacgggttggc | aaacgccaacccgtcccaggcccc |
| 39 | Rv0774c | gggagcacggtccttggcgctacc | aaacggtagcgccaaggaccgtgc |
| 40 | Rv0794c | gggactcggcgcgggacccgttgg | aaacccaacgggtcccgcgccgag |
| 41 | Rv0800 | gggacgggctggctcgctggtggc | aaacgccaccagcgagccagcccg |
| 42 | Rv0806c | gggactggaatccggcctgacccc | aaacggggtcaggccggattccag |
| 43 | Rv0838 | gggatcggccccacgacgttcagg | aaaccctgaacgtcgtggggccga |
| 44 | Rv0861c | gggaagtccgataagacggtgctg | aaaccagcaccgtcttatcggact |
| 45 | Rv0862c | gggaccttgcccagccaccacccg | aaaccgggtggtggctgggcaagg |
| 46 | Rv0878c | gggagtgctctcgaatctgtttgg | aaacccaaacagattcgagagcac |
| 47 | Rv0887c | gggagatgacgcccacctcgctgg | aaacccagcgaggtgggcgtcatc |
| 48 | Rv0907 | gggaggcctggtcggcgagctgat | aaacatcagctcgccgaccaggcc |
| 49 | Rv0911 | gggagttcgtcgtgcctggacccg | aaaccgggtccaggcacgacgaac |
| 50 | Rv0913c | gggaggcatctacctgcgcaacac | aaacgtgttgcgcaggtagatgcc |
| 51 | Rv0920c | gggagtgctcgaagccgccctgca | aaactgcagggcggcttcgagcac |
| 52 | Rv0922 | gggattgaagtcgctgcgatgggc | aaacgcccatcgcagcgacttcaa |
| 53 | Rv0931c | gggataacgcaccgcgacgtaaaa | aaacttttacgtcgcggtgcgtta |
| 54 | Rv0938 | gggagaccaacccgcgttcttcga | aaactcgaagaacgcgggttggtc |
| 55 | Rv0977 | gggaaatggcgtgctgttgaagac | aaacgtcttcaacagcacgccatt |
| 56 | Rv0980c | gggagcgcgcaggtcgcggcctac | aaacgtaggccgcgacctgcgcgc |
| 57 | Rv0988 | gggattagttggcgttttggtcga | aaactcgaccaaaacgccaactaa |
| 58 | Rv1050 | gggaaccggtgcctccagcggcat | aaacatgccgctggaggcaccggt |
| 59 | Rv1057 | gggatcgcgatcgccatgggcaat | aaacattgcccatggcgatcgcga |
| 60 | Rv1073 | gggaaacgatgttagggagctcgc | aaacgcgagctccctaacatcgtt |
| 61 | Rv1079 | gggagaggcctcgctggcggcagt | aaacactgccgccagcgaggcctc |
| 62 | Rv1084 | gggagtctgtatcaaggtcgaccg | aaaccggtcgaccttgatacagac |
| 63 | Rv1090 | gggaccagattttgtcggcgccga | aaactcggcgccgacaaaatctgg |
| 64 | Rv1124 | gggaatgcgggctggacgcggcga | aaactcgccgcgtccagcccgcat |
| 65 | Rv1135c | gggaatgccagctggctaagcacg | aaaccgtgcttagccagctggcat |
| 66 | Rv1162 | gggacatacctgctcggtgacctg | aaaccaggtcaccgagcaggtatg |
| 67 | Rv1243c | gggagccatcgcgtcgctgttttc | aaacgaaaacagcgacgcgatggc |
| 68 | Rv1259 | gggatgcgctgccgcagcccggat | aaacatccgggctgcggcagcgca |
| 69 | Rv1266c | gggatacgaggccgagcacaccgt | aaacacggtgtgctcggcctcgta |
| 70 | Rv1268c | gggagccgccgccaagtactggcg | aaaccgccagtacttggcggcggc |
| 71 | Rv1317c | gggaccgacgcctcgcctgggtct | aaacagacccaggcgaggcgtcgg |
| 72 | Rv1377c | gggagacaacgcgatctacctcgc | aaacgcgaggtagatcgcgttgtc |
| 73 | Rv1386 | gggacaggcgccagcgctgccatc | aaacgatggcagcgctggcgcctg |
| 74 | Rv1407 | gggagtcgaccaccgtcgagcagg | aaaccctgctcgacggtggtcgac |
| 75 | Rv1426c | gggatttggttgggctggccgacg | aaaccgtcggccagcccaaccaaa |
| 76 | Rv1454c | gggaacgccgaattctgcacagcg | aaaccgctgtgcagaattcggcgt |
| 77 | Rv1468c | gggagagttcgtgtcgggagcggc | aaacgccgctcccgacacgaactc |
| 78 | Rv1515c | gggagggctaaccaagtgatggca | aaactgccatcacttggttagccc |
| 79 | Rv1548c | gggaaccacggccgcagcgcaggc | aaacgcctgcgctgcggccgtggt |
| 80 | Rv1635c | gggaagtcgccgaaccgccggtga | aaactcaccggcggttcggcgact |
| 81 | Rv1665 | gggatttcccggccttaaggaaca | aaactgttccttaaggccgggaaa |
| 82 | Rv1677 | gggattgatgggcacgactttcac | aaacgtgaaagtcgtgcccatcaa |
| 83 | Rv1679 | gggatggccacaccgggcgttgtg | aaaccacaacgcccggtgtggcca |
| 84 | Rv1688 | gggactacttagggccgccgccat | aaacatggcggcggccctaagtag |
| 85 | Rv1743 | gggacccgcaccgcggggcgcctg | aaaccaggcgccccgcggtgcggg |
| 86 | Rv1746 | gggacggcatcccagactgccccg | aaaccggggcagtctgggatgccg |
| 87 | Rv1753c | gggatgatattcgccggggcaggg | aaacccctgccccggcgaatatca |
| 88 | Rv1754c | gggagtcaattgcaacgtccagcc | aaacggctggacgttgcaattgac |
| 89 | Rv1758 | gggagcgctgcgttccaagattgg | aaacccaatcttggaacgcagcgc |
| 90 | Rv1771 | gggaatatggagtaattggcctgg | aaacccaggccaattactccatat |
| 91 | Rv1789 | gggaatggtcgctgcggcgtcggc | aaacgccgacgccgcagcgaccat |
| 92 | Rv1807 | gggacagcctggcacggcttgtcc | aaacggacaagccgtgccaggctg |
| 93 | Rv1812c | gggacacggtcgacgtcatggtgg | aaacccaccatgacgtcgaccgtg |
| 94 | Rv1819c | gggaagccgtccatcgattggtct | aaacagaccaatcgatggacggct |
| 95 | Rv1869c | gggatacgaccggccgccgctttc | aaacgaaagcggcggccggtcgta |
| 96 | Rv1911c | gggagtacgtgcactggatcgtga | aaactcacgatccagtgcacgtac |
| 97 | Rv1917c | gggatcatattcggcggggcggga | aaactcccgccccgccgaatatga |
| 98 | Rv1918c | gggagccgcggcggcgacccaggc | aaacgcctgggtcgccgccgcggc |
| 99 | Rv1938 | gggatgcaaaaggcctaccgcatc | aaacgatgcggtaggccttttgca |
| 100 | Rv1966 | gggaatcggtcggatcgagtgggc | aaacgcccactcgatccgaccgat |
| 101 | Rv1968 | gggacgaggtctccggctatccgg | aaacccggatagccggagacctcg |
| 102 | Rv1984c | gggagggtctacgcggtgaactac | aaacgtagttcaccgcgtagaccc |
| 103 | Rv1997 | gggaacggcgaggccgcccaacga | aaactcgttgggcggcctcgccgt |
| 104 | Rv2006 | gggaagccgacttcttggccgccc | aaacgggcggccaagaagtcggct |
| 105 | Rv2030c | gggagcgaggccggccgggtgctg | aaaccagcacccggccggcctcgc |
| 106 | Rv2072c | gggattggcgtgcgcccggatggg | aaaccccatccgggcgcacgccaa |
| 107 | Rv2088 | gggaagagcggatgctaggtgccg | aaaccggcacctagcatccgctct |
| 108 | Rv2092c | gggaccgagctggaccggttcacc | aaacggtgaaccggtccagctcgg |
| 109 | Rv2114 | gggagcttgtccgggcaacgttac | aaacgtaacgttgcccggacaagc |
| 110 | Rv2227 | gggatcccgagtgatcgagcgtct | aaacagacgctcgatcactcggga |
| 111 | Rv2234 | gggaatctgccggtcgccaatggc | aaacgccattggcgaccggcagat |
| 112 | Rv2258c | gggagcggagcactcgagaccacc | aaacggtggtctcgagtgctccgc |
| 113 | Rv2260 | gggatcgaactcgatggcggcagt | aaacactgccgccatcgagttcga |
| 114 | Rv2284 | gggaacgtcccgatctagatctga | aaactcagatctagatcgggacgt |
| 115 | Rv2295 | gggagtcgaagagacctccacccc | aaacggggtggaggtctcttcgac |
| 116 | Rv2296 | gggatccgacaagccgactcgcat | aaacatgcgagtcggcttgtcgga |
| 117 | Rv2320c | gggacgacaacgtcgatgagcctt | aaacaaggctcatcgacgttgtcg |
| 118 | Rv2340c | gggaccgctgcggccgccccgacg | aaaccgtcggggcggccgcagcgg |
| 119 | Rv2430c | gggagggcgtggaggtcgttggat | aaacatccaacgacctccacgccc |
| 120 | Rv2521 | gggaacgtgtcgctggccgactac | aaacgtagtcggccagcgacacgt |
| 121 | Rv2577 | gggaggtctgcacctgcagttcgg | aaacccgaactgcaggtgcagacc |
| 122 | Rv2601 | gggacgggctacatagccgcgctg | aaaccagcgcggctatgtagcccg |
| 123 | Rv2641 | gggaccgcttaagttggtgctgct | aaacagcagcaccaacttaagcgg |
| 124 | Rv2765 | gggaagaccgcgttcggcgatccg | aaaccggatcgccgaacgcggtct |
| 125 | Rv2790c | gggagctacgtcggctacgtctac | aaacgtagacgtagccgacgtagc |
| 126 | Rv2793c | gggacgtggttatcgacaagcccg | aaaccgggcttgtcgataaccacg |
| 127 | Rv2812 | gggagtggcggtcggcgatgacga | aaactcgtcatcgccgaccgccac |
| 128 | Rv2854 | gggagtcggccgcggtccgcaaac | aaacgtttgcggaccgcggccgac |
| 129 | Rv2855 | gggacatcggaaccggttcgggca | aaactgcccgaaccggttccgatg |
| 130 | Rv2914c | gggaacggatcctgttggccgact | aaacagtcggccaacaggatccgt |
| 131 | Rv3060c | gggatccgccgcggctggccgatc | aaacgatcggccagccgcggcgga |
| 132 | Rv3061c | gggagctcgcgcgttcttgacttc | aaacgaagtcaagaacgcgcgagc |
| 133 | Rv3080c | gggagaggctggattcgacaatgt | aaacacattgtcgaatccagcctc |
| 134 | Rv3139 | gggactgctaaccccgattgtcgg | aaacccgacaatcggggttagcag |
| 135 | Rv3153 | gggaagacggtcaccgaggagtat | aaacatactcctcggtgaccgtct |
| 136 | Rv3159c | gggagtgttgtcgaatctgtttgg | aaacccaaacagattcgacaacac |
| 137 | Rv3253c | gggagtcgacggcgcatgaagtcg | aaaccgacttcatgcgccgtcgac |
| 138 | Rv3274c | gggagatcggcgatacgggctttg | aaaccaaagcccgtatcgccgatc |
| 139 | Rv3283 | gggactggcgatcgtcgaatccga | aaactcggattcgacgatcgccag |
| 140 | Rv3304 | gggatacgggtcgaacatgcatcc | aaacggatgcatgttcgacccgta |
| 141 | Rv3310 | gggatggcggtgatcggtgtcctg | aaaccaggacaccgatcaccgcca |
| 142 | Rv3438 | gggatctcgctattcgggtctatt | aaacaatagacccgaatagcgaga |
| 143 | Rv3476c | gggagccgagacccgtcgggccat | aaacatggcccgacgggtctcggc |
| 144 | Rv3524 | gggagtcgtggtgccgatcgacct | aaacaggtcgatcggcaccacgac |
| 145 | Rv3533c | gggatccggggcctcggcggcggc | aaacgccgccgccgaggccccgga |
| 146 | Rv3537 | gggaggtctctcgacggtagtcgt | aaacacgactaccgtcgagagacc |
| 147 | Rv3548c | gggaatgatcgccaacaccagcga | aaactcgctggtgttggcgatcat |
| 148 | Rv3558 | gggaggacgggctggccgcggagt | aaacactccgcggccagcccgtcc |
| 149 | Rv3569c | gggatcgaatccacctcgcgcttt | aaacaaagcgcgaggtggattcga |
| 150 | Rv3699 | gggagactgggacagcgcctaccg | aaaccggtaggcgctgtcccagtc |
| 151 | Rv3707c | gggaaatcggtccgacggccggta | aaactaccggccgtcggaccgatt |
| 152 | Rv3724B | gggacttccctaagacggtcatcg | aaaccgatgaccgtcttagggaag |
| 153 | Rv3725 | gggatctcggtcaagccgtcgagc | aaacgctcgacggcttgaccgaga |
| 154 | Rv3730c | gggaggtgcggttgactagcccgg | aaacccgggctagtcaaccgcacc |
| 155 | Rv3774 | gggagaatccgtcaccgtcgaaac | aaacgtttcgacggtgacggattc |
| 156 | Rv3786c | gggatgattcctgagtccgccggg | aaaccccggcggactcaggaatca |
| 157 | Rv3811 | gggaacgcccaacacccagctcgc | aaacgcgagctgggtgttgggcgt |
| 158 | Rv3852 | gggagacgcatcgacgccgccagc | aaacgctggcggcgtcgatgcgtc |
| 159 | Rv3873 | gggaccgcgcgcctgaactctctg | aaaccagagagttcaggcgcgcgg |

| **Table S2. Top 10 differentially expressed genes and their functional annotations in WT- and ΔRv0066c-infected groups** | | | | | | | | | | | | | | | | | | | | | | | | | | | | | | | | | |
| --- | --- | --- | --- | --- | --- | --- | --- | --- | --- | --- | --- | --- | --- | --- | --- | --- | --- | --- | --- | --- | --- | --- | --- | --- | --- | --- | --- | --- | --- | --- | --- | --- | --- |
| geneID | length | T-wt-1.rawreads | T-wt-1.FPKM | T-wt-2.rawreads | T-wt-2.FPKM | T-wt-3.rawreads | T-wt-3.FPKM | T-ΔRv0066c-1.rawreads | T-ΔRv0066c-1.FPKM | T-ΔRv0066c-2.rawreads | T-ΔRv0066c-2.FPKM | T-ΔRv0066c-3.rawreads | T-ΔRv0066c-3.FPKM | baseMean | foldchange | log2FoldChange | lfcSE | stat | pvalue | padj | Regulation | Chr | Start | End | Strand | GeneSymbol | Description | | GO_BP | GO_CC | GO_MF | KO | Pathway |
| ENSG00000171195 | 2798 | 4 | 0.07 | 2 | 0.04 | 1 | 0.02 | 2 | 0.04 | 137 | 3.08 | 154 | 3.04 | 53.8326585759196 | 48.5418034406119 | 5.60115580383984 | 1.51614517290675 | 3.69434003018412 | 0.000220458630611561 | 0.329711422518395 | Ups | 4 | 70430492 | 70482997 | + | MUC7 | mucin 7%2C secreted [Source:HGNC Symbol%3BAcc:HGNC:7518] | | GO:0031640:killing of cells of other organism; | GO:0070062:extracellular exosome;GO:0005796:Golgi lumen;GO:0005886:plasma membrane; | - | K13909 | ;ko04970 Salivary secretion; |
| ENSG00000146677 | 408 | 1 | 0.12 | 1 | 0.12 | 1 | 0.13 | 7 | 0.94 | 5 | 0.77 | 3 | 0.41 | 3.11421184278001 | 5.61911089094527 | 2.49034187160274 | 1.20743503283457 | 2.0625058938007 | 0.0391595970138469 | 0.999012462187442 | Ups | 7 | 44467897 | 44468304 | + | RPL32P18 | ribosomal protein L32 pseudogene 18 [Source:HGNC Symbol%3BAcc:HGNC:36301] | | GO:0006412:translation; | GO:0022625:cytosolic large ribosomal subunit; | GO:0003735:structural constituent of ribosome; | K02912 | ;ko03010 Ribosome;ko05171 Coronavirus disease - COVID-19; |
| ENSG00000207956 | 98 | 2 | 1.02 | 3 | 1.52 | 1 | 0.54 | 7 | 3.91 | 7 | 4.49 | 16 | 9.01 | 6.15672080737344 | 5.59533699705708 | 2.48422502473907 | 0.859130502653432 | 2.89155723963533 | 0.0038333775614959 | 0.976667254675368 | Ups | 5 | 32394378 | 32394475 | - | MIR579 | microRNA 579 [Source:HGNC Symbol%3BAcc:HGNC:32835] | | - | - | - | - | - |
| ENSG00000254154 | 7841 | 1 | 0.01 | 2 | 0.01 | 2 | 0.01 | 13 | 0.09 | 6 | 0.05 | 6 | 0.04 | 5.13729631055825 | 5.5624529145416 | 2.47572121878755 | 0.934904435947006 | 2.64810083640237 | 0.00809453770648297 | 0.999012462187442 | Ups | 1 | 177928788 | 178038007 | - | CRYZL2P-SEC16B | CRYZL2P-SEC16B readthrough [Source:HGNC Symbol%3BAcc:HGNC:53757] | | - | - | - | - | - |
| ENSG00000240137 | 5773 | 2 | 0.02 | 1 | 0.01 | 2 | 0.02 | 6 | 0.06 | 14 | 0.15 | 4 | 0.04 | 5.1656229760345 | 5.51884738533679 | 2.46436699071858 | 0.960700457772921 | 2.56517728369926 | 0.0103123100289201 | 0.999012462187442 | Ups | 3 | 150703486 | 150723005 | + | ERICH6-AS1 | ERICH6 antisense RNA 1 [Source:HGNC Symbol%3BAcc:HGNC:41205] | | - | - | - | - | - |
| ENSG00000211452 | 2282 | 3 | 0.07 | 5 | 0.11 | 3 | 0.07 | 13 | 0.31 | 10 | 0.28 | 12 | 0.29 | 7.85170162796214 | 3.5680289865862 | 1.83512733572747 | 0.682080174206808 | 2.69048625824896 | 0.0071347973411626 | 0.999012462187442 | Ups | 1 | 53891239 | 53911086 | + | DIO1 | iodothyronine deiodinase 1 [Source:HGNC Symbol%3BAcc:HGNC:2883] | | GO:0006520:cellular amino acid metabolic process;GO:0042446:hormone biosynthetic process;GO:0006590:thyroid hormone generation;GO:0042403:thyroid hormone metabolic process;GO:0006091:generation of precursor metabolites and energy; | GO:0005789:endoplasmic reticulum membrane;GO:0016021:integral component of membrane;GO:0005886:plasma membrane; | GO:0008430:selenium binding;GO:0004800:thyroxine 5'-deiodinase activity; | K01562 | ;ko04919 Thyroid hormone signaling pathway; |
| ENSG00000215270 | 1049 | 3 | 0.14 | 3 | 0.14 | 2 | 0.1 | 3 | 0.16 | 14 | 0.84 | 6 | 0.32 | 5.46010332368307 | 3.32649061354972 | 1.73400096283342 | 0.870561576169787 | 1.99181885612562 | 0.0463909365816142 | 0.999012462187442 | Ups | 22 | 15854195 | 15855243 | - | TOMM40P2 | TOMM40 pseudogene 2 [Source:HGNC Symbol%3BAcc:HGNC:54624] | | GO:0030150:protein import into mitochondrial matrix;GO:0006626:protein targeting to mitochondrion; | GO:0005829:cytosol;GO:0032592:integral component of mitochondrial membrane;GO:0044233:ER-mitochondrion membrane contact site;GO:0005743:mitochondrial inner membrane;GO:0005742:mitochondrial outer membrane translocase complex; | GO:0008320:protein transmembrane transporter activity; | K11518 | ;ko05014 Amyotrophic lateral sclerosis;ko05022 Pathways of neurodegeneration - multiple diseases; |
| ENSG00000225675 | 567 | 4 | 0.35 | 3 | 0.26 | 2 | 0.19 | 12 | 1.16 | 11 | 1.22 | 3 | 0.29 | 6.03800097064811 | 3.28302055077233 | 1.71502378079866 | 0.812776748906341 | 2.11007977665007 | 0.0348514849049755 | 0.999012462187442 | Ups | 1 | 53328233 | 53336509 | + | LRP8-DT | LRP8 divergent transcript [Source:HGNC Symbol%3BAcc:HGNC:52561] | | - | - | - | - | - |
| ENSG00000224389 | 7174 | 5 | 0.03 | 2 | 0.01 | 3 | 0.02 | 14 | 0.11 | 5 | 0.04 | 10 | 0.08 | 6.56605829269487 | 3.21510596234835 | 1.68486628618007 | 0.756900806509222 | 2.22600672596792 | 0.0260137309015394 | 0.999012462187442 | Ups | 6 | 32014795 | 32035418 | + | C4B | complement C4B (Chido blood group) [Source:HGNC Symbol%3BAcc:HGNC:1324] | | GO:0006956:complement activation;GO:0006958:complement activation, classical pathway;GO:0032490:detection of molecule of bacterial origin;GO:0006954:inflammatory response;GO:0045087:innate immune response;GO:0008228:opsonization;GO:2000427:positive regulation of apoptotic cell clearance; | GO:0030424:axon;GO:0072562:blood microparticle;GO:0030425:dendrite;GO:0070062:extracellular exosome;GO:0005576:extracellular region;GO:0005615:extracellular space;GO:0005886:plasma membrane;GO:0045202:synapse;GO:0005788:endoplasmic reticulum lumen;GO:0043025:neuronal cell body; | GO:0030246:carbohydrate binding;GO:0001848:complement binding;GO:0004866:endopeptidase inhibitor activity;GO:0001849:complement component C1q binding; | K03989 | ;ko04610 Complement and coagulation cascades;ko05133 Pertussis;ko05150 Staphylococcus aureus infection;ko05171 Coronavirus disease - COVID-19;ko05322 Systemic lupus erythematosus; |
| ENSG00000152154 | 2472 | 5 | 0.1 | 3 | 0.06 | 2 | 0.04 | 7 | 0.16 | 5 | 0.13 | 14 | 0.31 | 6.05746767453824 | 2.89975565324356 | 1.53593133723823 | 0.782502664903823 | 1.96284486446702 | 0.0496642029262475 | 0.999012462187442 | Ups | 2 | 39664982 | 39717963 | + | TMEM178A | transmembrane protein 178A [Source:HGNC Symbol%3BAcc:HGNC:28517] | | GO:0045671:negative regulation of osteoclast differentiation;GO:0051480:cytosolic calcium ion homeostasis; | GO:0005789:endoplasmic reticulum membrane;GO:0016021:integral component of membrane;GO:0016020:membrane; | - | - | - |
| ENSG00000130812 | 2063 | 26 | 0.63 | 16 | 0.39 | 11 | 0.28 | 3 | 0.08 | 5 | 0.15 | 1 | 0.03 | 9.87418414125381 | 0.193492720993697 | -2.3696488 | 0.690265204022781 | -3.432954155 | 0.000597043041593629 | 0.486747194118311 | Down | 19 | 10092338 | 10102678 | - | ANGPTL6 | angiopoietin like 6 [Source:HGNC Symbol%3BAcc:HGNC:23140] | | GO:0001525:angiogenesis;GO:0030154:cell differentiation; | GO:0070062:extracellular exosome;GO:0005615:extracellular space;GO:0030141:secretory granule; | GO:0005102:receptor binding; | - | - |
| ENSG00000137078 | 1277 | 8 | 0.31 | 18 | 0.7 | 10 | 0.41 | 2 | 0.09 | 4 | 0.2 | 1 | 0.04 | 6.89637968098502 | 0.221150207239326 | -2.176901501 | 0.801688230931491 | -2.715396605 | 0.00661964430108911 | 0.999012462187442 | Down | 9 | 35649295 | 35650931 | - | SIT1 | signaling threshold regulating transmembrane adaptor 1 [Source:HGNC Symbol%3BAcc:HGNC:17710] | | GO:0002250:adaptive immune response;GO:0050863:regulation of T cell activation;GO:0007165:signal transduction;GO:0043029:T cell homeostasis; | GO:0070062:extracellular exosome;GO:0005887:integral component of plasma membrane;GO:0005886:plasma membrane; | GO:0019900:kinase binding;GO:0042169:SH2 domain binding; | - | - |
| ENSG00000163888 | 1452 | 9 | 0.31 | 7 | 0.24 | 11 | 0.4 | 2 | 0.08 | 2 | 0.09 | 2 | 0.08 | 5.31396455358784 | 0.2485855287777 | -2.008185781 | 0.871890719538107 | -2.303253993 | 0.0212645565305993 | 0.999012462187442 | Down | 3 | 184259213 | 184261553 | - | CAMK2N2 | calcium/calmodulin dependent protein kinase II inhibitor 2 [Source:HGNC Symbol%3BAcc:HGNC:24197] | | GO:0006469:negative regulation of protein kinase activity; | GO:0005813:centrosome;GO:0005829:cytosol;GO:0005654:nucleoplasm; | GO:0008427:calcium-dependent protein kinase inhibitor activity;GO:0019901:protein kinase binding; | K01415 | - |
| ENSG00000220785 | 3496 | 12 | 0.17 | 7 | 0.1 | 7 | 0.11 | 4 | 0.06 | 1 | 0.02 | 1 | 0.02 | 5.09173164230374 | 0.257628504291139 | -1.956635871 | 0.901597024086177 | -2.170188919 | 0.0299925371679106 | 0.999012462187442 | Down | 1 | 32231656 | 32241710 | - | MTMR9LP | myotubularin related protein 9 like%2C pseudogene [Source:HGNC Symbol%3BAcc:HGNC:27920] | | GO:0010507:negative regulation of autophagy;GO:0046856:phosphatidylinositol dephosphorylation; | - | GO:0004438:phosphatidylinositol-3-phosphatase activity; | K18084 | - |
| ENSG00000260001 | 2831 | 19 | 0.34 | 20 | 0.35 | 19 | 0.35 | 7 | 0.14 | 1 | 0.02 | 7 | 0.14 | 11.6404448195812 | 0.28674946431238 | -1.802137304 | 0.577246156729735 | -3.121956349 | 0.00179653573912184 | 0.741991532920393 | Down | 19 | 7914830 | 7919097 | + | TGFBR3L | transforming growth factor beta receptor 3 like [Source:HGNC Symbol%3BAcc:HGNC:44152] | | GO:0001525:angiogenesis;GO:0016477:cell migration;GO:0001837:epithelial to mesenchymal transition;GO:0017015:regulation of transforming growth factor beta receptor signaling pathway;GO:0007179:transforming growth factor beta receptor signaling pathway;GO:0001570:vasculogenesis; | GO:0009986:cell surface;GO:0005615:extracellular space;GO:0016021:integral component of membrane; | GO:0005539:glycosaminoglycan binding;GO:0005024:transforming growth factor beta-activated receptor activity;GO:0005114:type II transforming growth factor beta receptor binding; | K15209;K17985 | -;ko04137 Mitophagy - animal;ko04140 Autophagy - animal;ko05010 Alzheimer disease;ko05014 Amyotrophic lateral sclerosis;ko05016 Huntington disease;ko05017 Spinocerebellar ataxia;ko05022 Pathways of neurodegeneration - multiple diseases; |
| ENSG00000266920 | 1099 | 8 | 0.36 | 15 | 0.68 | 9 | 0.43 | 2 | 0.1 | 3 | 0.17 | 5 | 0.25 | 6.75695588423782 | 0.35134747317769 | -1.509029572 | 0.739827179999274 | -2.039705505 | 0.0413796662877709 | 0.999012462187442 | Down | 18 | 62442028 | 62443126 | + | ACTBP9 | ACTB pseudogene 9 [Source:HGNC Symbol%3BAcc:HGNC:142] | | - | GO:0015629:actin cytoskeleton;GO:0005856:cytoskeleton;GO:0097433:dense body;GO:0005925:focal adhesion;GO:0005634:nucleus;GO:0005886:plasma membrane;GO:0032991:macromolecular complex; | GO:0005524:ATP binding; | K05692 | ;ko04015 Rap1 signaling pathway;ko04145 Phagosome;ko04210 Apoptosis;ko04390 Hippo signaling pathway;ko04391 Hippo signaling pathway - fly;ko04510 Focal adhesion;ko04520 Adherens junction;ko04530 Tight junction;ko04611 Platelet activation;ko04613 Neutrophil extracellular trap formation;ko04670 Leukocyte transendothelial migration;ko04714 Thermogenesis;ko04745 Phototransduction - fly;ko04810 Regulation of actin cytoskeleton;ko04919 Thyroid hormone signaling pathway;ko04921 Oxytocin signaling pathway;ko04971 Gastric acid secretion;ko05014 Amyotrophic lateral sclerosis;ko05100 Bacterial invasion of epithelial cells;ko05110 Vibrio cholerae infection;ko05130 Pathogenic Escherichia coli infection;ko05131 Shigellosis;ko05132 Salmonella infection;ko05135 Yersinia infection;ko05164 Influenza A;ko05205 Proteoglycans in cancer;ko05225 Hepatocellular carcinoma;ko05410 Hypertrophic cardiomyopathy;ko05412 Arrhythmogenic right ventricular cardiomyopathy;ko05414 Dilated cardiomyopathy;ko05416 Viral myocarditis;ko05418 Fluid shear stress and atherosclerosis |
| ENSG00000233868 | 390 | 17 | 2.18 | 20 | 2.55 | 10 | 1.36 | 5 | 0.7 | 5 | 0.81 | 5 | 0.71 | 9.95433454712327 | 0.360347035671633 | -1.472541118 | 0.59903975403472 | -2.458169274 | 0.0139647334263109 | 0.999012462187442 | Down | 2 | 221944223 | 221944612 | + | LLPHP3 | LLPH pseudogene 3 [Source:HGNC Symbol%3BAcc:HGNC:54775] | | GO:0097484:dendrite extension;GO:0060999:positive regulation of dendritic spine development; | GO:0005694:chromosome;GO:0005730:nucleolus; | GO:0001099:basal RNA polymerase II transcription machinery binding;GO:0003723:RNA binding; | - | - |
| ENSG00000119737 | 2094 | 14 | 0.33 | 29 | 0.69 | 21 | 0.53 | 5 | 0.13 | 6 | 0.18 | 10 | 0.26 | 13.7139192576059 | 0.367791630546782 | -1.443039445 | 0.520195820721531 | -2.774031215 | 0.00553663654161114 | 0.999012462187442 | Down | 2 | 53852912 | 53859967 | - | GPR75 | G protein-coupled receptor 75 [Source:HGNC Symbol%3BAcc:HGNC:4526] | | GO:0070098:chemokine-mediated signaling pathway;GO:0007186:G-protein coupled receptor signaling pathway;GO:1901214:regulation of neuron death; | GO:0005887:integral component of plasma membrane;GO:0005886:plasma membrane; | GO:0016493:C-C chemokine receptor activity;GO:0004930:G-protein coupled receptor activity; | K08417 | - |
| ENSG00000105146 | 1741 | 17 | 0.49 | 12 | 0.34 | 21 | 0.64 | 4 | 0.13 | 5 | 0.18 | 8 | 0.25 | 10.8733075587549 | 0.379617676975371 | -1.397380922 | 0.575150783576405 | -2.429590573 | 0.015115887598007 | 0.999012462187442 | Down | 19 | 57231009 | 57235548 | + | AURKC | aurora kinase C [Source:HGNC Symbol%3BAcc:HGNC:11391] | | GO:0008608:attachment of spindle microtubules to kinetochore;GO:0051301:cell division;GO:0016570:histone modification;GO:0051321:meiotic cell cycle;GO:0051256:mitotic spindle midzone assembly;GO:0007052:mitotic spindle organization;GO:0048599:oocyte development;GO:0032467:positive regulation of cytokinesis;GO:0006468:protein phosphorylation;GO:0032465:regulation of cytokinesis;GO:0007283:spermatogenesis;GO:0009838:abscission;GO:0034644:cellular response to UV;GO:0036089:cleavage furrow formation;GO:0043988:histone H3-S28 phosphorylation;GO:0044878:cytokinesis checkpoint;GO:0007094:mitotic spindle assembly checkpoint;GO:0002903:negative regulation of B cell apoptotic process;GO:0032466:negative regulation of cytokinesis;GO:0000122:negative regulation of transcription from RNA polymerase II promoter;GO:0034501:protein localization to kinetochore; | GO:0032133:chromosome passenger complex;GO:0000775:chromosome, centromeric region;GO:0000793:condensed chromosome;GO:0005737:cytoplasm;GO:0030496:midbody;GO:0005634:nucleus;GO:0005819:spindle;GO:0005876:spindle microtubule;GO:0051233:spindle midzone;GO:0031616:spindle pole centrosome;GO:0005694:chromosome;GO:0070938:contractile ring;GO:0042585:germinal vesicle;GO:1990385:meiotic spindle midzone; | GO:0005524:ATP binding;GO:0035174:histone serine kinase activity;GO:0004672:protein kinase activity;GO:0004712:protein serine/threonine/tyrosine kinase activity;GO:0046872:metal ion binding;GO:0004674:protein serine/threonine kinase activity; | K11480 | - |
| ENSG00000213881 | 871 | 11 | 0.63 | 14 | 0.8 | 13 | 0.79 | 2 | 0.13 | 3 | 0.22 | 8 | 0.51 | 8.22707091558511 | 0.38215776198635 | -1.387759762 | 0.666383287169858 | -2.08252486 | 0.0372945545797985 | 0.999012462187442 | Down | 8 | 61202350 | 61203220 | + | NPM1P6 | nucleophosmin 1 pseudogene 6 [Source:HGNC Symbol%3BAcc:HGNC:7926] | GO:0006338:chromatin remodeling;GO:0006281:DNA repair;GO:0043066:negative regulation of apoptotic process;GO:0044387:negative regulation of protein kinase activity by regulation of protein phosphorylation;GO:1902751:positive regulation of cell cycle G2/M phase transition;GO:0008284:positive regulation of cell proliferation;GO:0045944:positive regulation of transcription from RNA polymerase II promoter;GO:0045893:positive regulation of transcription, DNA-templated;GO:0045727:positive regulation of translation;GO:0046599:regulation of centriole replication;GO:0010824:regulation of centrosome duplication;GO:0060735:regulation of eIF2 alpha phosphorylation by dsRNA;GO:0032071:regulation of endodeoxyribonuclease activity;GO:0060699:regulation of endoribonuclease activity;GO:0042273:ribosomal large subunit biogenesis;GO:0000055:ribosomal large subunit export from nucleus;GO:0042274:ribosomal small subunit biogenesis;GO:0000056:ribosomal small subunit export from nucleus;GO:0006407:rRNA export from nucleus; | | GO:0005813:centrosome;GO:0005737:cytoplasm;GO:0005730:nucleolus;GO:0005654:nucleoplasm; | GO:0003682:chromatin binding;GO:0042393:histone binding;GO:0004860:protein kinase inhibitor activity;GO:0003723:RNA binding; | K11276 | - |

**Table S3. Bacterial strains**

| **Name** | **Description** | **Source** |
| --- | --- | --- |
| WT | H37Rv | This study |
| ΔRv0066c | H37Rv strain with deletion of Rv0066c of 2017 bp | This study |
| ΔRv3139 | H37Rv strain with deletion of Rv3139 of 1193 bp | This study |
| *E.coli* DH5α | *Escherichia coli* competent cells | Vazyme |
| ΔRv0062 - 1 | *M.tb* Rv0062 delete 4 bp | This study |
| ΔRv0062 - 2 | *M.tb* Rv0062 insert 2 bp | This study |
| ∆Rv0066c - 1 | *M.tb* Rv0066c delete 5 bp | This study |
| ∆Rv0066c - 2 | *M.tb* Rv0066c insert 1 bp | This study |
| ∆Rv0089 - 1 | *M.tb* Rv0089 delete 8 bp | This study |
| ∆Rv0089 - 2 | *M.tb* Rv0089 delete 2 bp | This study |
| ∆Rv0116c - 1 | *M.tb* Rv0116c delete 590 bp | This study |
| ∆Rv0116c - 2 | *M.tb* Rv0116c delete 7 bp | This study |
| ∆Rv0139 - 1 | *M.tb* Rv0139 insert 1 bp | This study |
| ∆Rv0139 - 2 | *M.tb* Rv0139 insert 1 bp | This study |
| ∆Rv0153c - 1 | *M.tb* Rv0153c delete 8 bp | This study |
| ∆Rv0153c - 2 | *M.tb* Rv0153c delete 10 bp | This study |
| ∆Rv0169 - 1 | *M.tb* Rv0169 insert 1 bp | This study |
| ∆Rv0169 - 2 | *M.tb* Rv0169 delete 1040 bp | This study |
| ∆Rv0183 - 1 | *M.tb* Rv0183 delete 8 bp | This study |
| ∆Rv0183 - 2 | *M.tb* Rv0183 delete 4 bp | This study |
| ∆Rv0187 - 1 | *M.tb* Rv0187 delete 5 bp | This study |
| ∆Rv0187 - 2 | *M.tb* Rv0187 insert 1 bp | This study |
| ∆Rv0198c - 1 | *M.tb* Rv0198c delete 14 bp | This study |
| ∆Rv0198c - 2 | *M.tb* Rv0198c insert 1 bp | This study |
| ∆Rv0229c - 1 | *M.tb* Rv0229c indelete 7 bp | This study |
| ∆Rv0229c - 2 | *M.tb* Rv0229c delete 1 bp | This study |
| ∆Rv0260c - 1 | *M.tb* Rv0260c delete 13 bp | This study |
| ∆Rv0260c - 2 | *M.tb* Rv0260c insert 1 bp | This study |
| ∆Rv0274 - 1 | *M.tb* Rv0274 insert 20 bp | This study |
| ∆Rv0274 - 2 | *M.tb* Rv0274 insert 323 bp | This study |
| ∆Rv0305c - 1 | *M.tb* Rv0305c delete 17 bp | This study |
| ∆Rv0305c - 2 | *M.tb* Rv0305c insert 1 bp | This study |
| ∆Rv0386 - 1 | *M.tb* Rv0386 delete 1 bp | This study |
| ∆Rv0386 - 2 | *M.tb* Rv0386 delete 14 bp | This study |
| ∆Rv0425c - 1 | *M.tb* Rv0425c delete 17 bp | This study |
| ∆Rv0425c - 2 | *M.tb* Rv0425c delete 2 bp | This study |
| ∆Rv0426c - 1 | *M.tb* Rv0426c insert 1 bp | This study |
| ∆Rv0426c - 2 | *M.tb* Rv0426c indelete 11 bp | This study |
| ∆Rv0432 - 1 | *M.tb* Rv0432 delete 10 bp | This study |
| ∆Rv0432 - 2 | *M.tb* Rv0432 delete 2 bp | This study |
| ∆Rv0435c - 1 | *M.tb* Rv0435c delete 2 bp | This study |
| ∆Rv0435c - 2 | *M.tb* Rv0435c insert 1 bp | This study |
| ∆Rv0446c - 1 | *M.tb* Rv0446c delete 8 bp | This study |
| ∆Rv0446c - 2 | *M.tb* Rv0446c insert 1 bp | This study |
| ∆Rv0518 - 1 | *M.tb* Rv0518 delete 8 bp | This study |
| ∆Rv0518 - 2 | *M.tb* Rv0518 insert 1 bp | This study |
| ∆Rv0561c - 1 | *M.tb* Rv0561c delete 1 bp | This study |
| ∆Rv0561c - 2 | *M.tb* Rv0561c insert 1 bp | This study |
| ∆Rv0571c - 1 | *M.tb* Rv0571c insert 1 bp | This study |
| ∆Rv0571c - 2 | *M.tb* Rv0571c insert 1 bp | This study |
| ∆Rv0577 - 1 | *M.tb* Rv0577 delete 13 bp | This study |
| ∆Rv0577 - 2 | *M.tb* Rv0577 delete 8 bp | This study |
| ∆Rv0594 - 1 | *M.tb* Rv0594 delete 16 bp | This study |
| ∆Rv0594 - 2 | *M.tb* Rv0594 delete 13 bp | This study |
| ∆Rv0629c - 1 | *M.tb* Rv0629c delete 8 bp | This study |
| ∆Rv0629c - 2 | *M.tb* Rv0629c delete 7 bp | This study |
| ∆Rv0648 - 1 | *M.tb* Rv0648 insert 1 bp | This study |
| ∆Rv0648 - 2 | *M.tb* Rv0648 insert 1 bp | This study |
| ∆Rv0654 - 1 | *M.tb* Rv0654 indelete 100 bp | This study |
| ∆Rv0654 - 2 | *M.tb* Rv0654 insert 115 bp | This study |
| ∆Rv0669c - 1 | *M.tb* Rv0669c delete 2 bp | This study |
| ∆Rv0669c - 2 | *M.tb* Rv0669c delete 5 bp | This study |
| ∆Rv0671 - 1 | *M.tb* Rv0671 delete 5 bp | This study |
| ∆Rv0671 - 2 | *M.tb* Rv0671 insert 1 bp | This study |
| ∆Rv0774c - 1 | *M.tb* Rv0774c delete 16 bp | This study |
| ∆Rv0774c - 2 | *M.tb* Rv0774c delete 8 bp | This study |
| ∆Rv0794c - 1 | *M.tb* Rv0794c delete 215 bp | This study |
| ∆Rv0794c - 2 | *M.tb* Rv0794c delete 10 bp | This study |
| ∆Rv0800 - 1 | *M.tb* Rv0800 delete 11 bp | This study |
| ∆Rv0800 - 2 | *M.tb* Rv0800 delete 7 bp | This study |
| ∆Rv0806c - 1 | *M.tb* Rv0806c delete 5 bp | This study |
| ∆Rv0806c - 2 | *M.tb* Rv0806c insert 1 bp | This study |
| ∆Rv0838 - 1 | *M.tb* Rv0838 delete 5 bp | This study |
| ∆Rv0838 - 2 | *M.tb* Rv0838 insert 1 bp | This study |
| ∆Rv0861c - 1 | *M.tb* Rv0861c delete 22 bp | This study |
| ∆Rv0861c - 2 | *M.tb* Rv0861c insert 2 bp | This study |
| ∆Rv0862c - 1 | *M.tb* Rv0862c delete 55 bp | This study |
| ∆Rv0862c - 2 | *M.tb* Rv0862c insert 1 bp | This study |
| ∆Rv0887c - 1 | *M.tb* Rv0887c delete 5 bp | This study |
| ∆Rv0887c - 2 | *M.tb* Rv0887c insert 13 bp | This study |
| ∆Rv0907 - 1 | *M.tb* Rv0907 delete 5 bp | This study |
| ∆Rv0907 - 2 | *M.tb* Rv0907 delete 16 bp | This study |
| ∆Rv0911 - 1 | *M.tb* Rv0911 delete 2 bp | This study |
| ∆Rv0911 - 2 | *M.tb* Rv0911 insert 1 bp | This study |
| ∆Rv0913c - 1 | *M.tb* Rv0913c delete 14 bp | This study |
| ∆Rv0913c - 2 | *M.tb* Rv0913c insert 1 bp | This study |
| ∆Rv0922 - 1 | *M.tb* Rv0922 delete 14 bp | This study |
| ∆Rv0922 - 2 | *M.tb* Rv0922 delete 1 bp | This study |
| ∆Rv0931c - 1 | *M.tb* Rv0931c delete 2 bp | This study |
| ∆Rv0931c - 2 | *M.tb* Rv0931c delete 5 bp | This study |
| ∆Rv0977 - 1 | *M.tb* Rv0977 delete 1 bp | This study |
| ∆Rv0977 - 2 | *M.tb* Rv0977 insert 1 bp | This study |
| ∆Rv0980c - 1 | *M.tb* Rv0980c delete 8 bp | This study |
| ∆Rv0980c - 2 | *M.tb* Rv0980c delete 13 bp | This study |
| ∆Rv0988 - 1 | *M.tb* Rv0988 delete 1 bp | This study |
| ∆Rv0988 - 2 | *M.tb* Rv0988 insert 1 bp | This study |
| ∆Rv1057 - 1 | *M.tb* Rv1057 insert 1 bp | This study |
| ∆Rv1057 - 2 | *M.tb* Rv1057 insert 35 bp | This study |
| ∆Rv1073 - 1 | *M.tb* Rv1073 insert 1 bp | This study |
| ∆Rv1073 - 2 | *M.tb* Rv1073 insert 1 bp | This study |
| ∆Rv1079 - 1 | *M.tb* Rv1079 delete 112 bp | This study |
| ∆Rv1079 - 2 | *M.tb* Rv1079 insert 1 bp | This study |
| ∆Rv1084 - 1 | *M.tb* Rv1084 delete 8 bp | This study |
| ∆Rv1084 - 2 | *M.tb* Rv1084 insert 1 bp | This study |
| ∆Rv1090 - 1 | *M.tb* Rv1090 insert 1 bp | This study |
| ∆Rv1090 - 2 | *M.tb* Rv1090 insert 1 bp | This study |
| ∆Rv1124 - 1 | *M.tb* Rv1124 delete 10 bp | This study |
| ∆Rv1124 - 2 | *M.tb* Rv1124 indelete 8 bp | This study |
| ∆Rv1135c - 1 | *M.tb* Rv1135c delete 4 bp | This study |
| ∆Rv1135c - 2 | *M.tb* Rv1135c insert 1 bp | This study |
| ∆Rv1162 - 1 | *M.tb* Rv1162 insert 1 bp | This study |
| ∆Rv1162 - 2 | *M.tb* Rv1162 insert 1 bp | This study |
| ∆Rv1243c - 1 | *M.tb* Rv1243c delete 8 bp | This study |
| ∆Rv1243c - 2 | *M.tb* Rv1243c delete 10 bp | This study |
| ∆Rv1259 - 1 | *M.tb* Rv1259 delete 5 bp | This study |
| ∆Rv1259 - 2 | *M.tb* Rv1259 insert 1 bp | This study |
| ∆Rv1266c - 1 | *M.tb* Rv1266c insert 1 bp | This study |
| ∆Rv1266c - 2 | *M.tb* Rv1266c insert 1 bp | This study |
| ∆Rv1268c - 1 | *M.tb* Rv1268c delete 5 bp | This study |
| ∆Rv1268c - 2 | *M.tb* Rv1268c delete 7 bp | This study |
| ∆Rv1317c - 1 | *M.tb* Rv1317c delete 11 bp | This study |
| ∆Rv1317c - 2 | *M.tb* Rv1317c delete 1 bp | This study |
| ∆Rv1377c - 1 | *M.tb* Rv1377c delete 10 bp | This study |
| ∆Rv1377c - 2 | *M.tb* Rv1377c delete 5 bp | This study |
| ∆Rv1386 - 1 | *M.tb* Rv1386 insert 1 bp | This study |
| ∆Rv1386 - 2 | *M.tb* Rv1386 insert 1 bp | This study |
| ∆Rv1407 - 1 | *M.tb* Rv1407 delete 11 bp | This study |
| ∆Rv1407 - 2 | *M.tb* Rv1407 delete 1 bp | This study |
| ∆Rv1426c - 1 | *M.tb* Rv1426c insert 1 bp | This study |
| ∆Rv1426c - 2 | *M.tb* Rv1426c insert 1 bp | This study |
| ∆Rv1454c - 1 | *M.tb* Rv1454c delete 7 bp | This study |
| ∆Rv1454c - 2 | *M.tb* Rv1454c insert 1 bp | This study |
| ∆Rv1468c - 1 | *M.tb* Rv1468c insert 1 bp | This study |
| ∆Rv1468c - 2 | *M.tb* Rv1468c insert 50 bp | This study |
| ∆Rv1515c - 1 | *M.tb* Rv1515c delete 4 bp | This study |
| ∆Rv1515c - 2 | *M.tb* Rv1515c indelete 2 bp | This study |
| ∆Rv1548c - 1 | *M.tb* Rv1548c delete 16 bp | This study |
| ∆Rv1548c - 2 | *M.tb* Rv1548c delete 1 bp | This study |
| ∆Rv1635c - 1 | *M.tb* Rv1635c delete 4 bp | This study |
| ∆Rv1635c - 2 | *M.tb* Rv1635c insert 1 bp | This study |
| ∆Rv1665 - 1 | *M.tb* Rv1665 delete 1 bp | This study |
| ∆Rv1665 - 2 | *M.tb* Rv1665 insert 1 bp | This study |
| ∆Rv1677 - 1 | *M.tb* Rv1677 delete 11 bp | This study |
| ∆Rv1677 - 2 | *M.tb* Rv1677 delete 7 bp | This study |
| ∆Rv1679 - 1 | *M.tb* Rv1679 delete 10 bp | This study |
| ∆Rv1679 - 2 | *M.tb* Rv1679 delete 17 bp | This study |
| ∆Rv1688 - 1 | *M.tb* Rv1688 insert 1 bp | This study |
| ∆Rv1688 - 2 | *M.tb* Rv1688 insert 1 bp | This study |
| ∆Rv1743 - 1 | *M.tb* Rv1743 delete 1 bp | This study |
| ∆Rv1743 - 2 | *M.tb* Rv1743 insert 1 bp | This study |
| ∆Rv1746 - 1 | *M.tb* Rv1746 delete 5 bp | This study |
| ∆Rv1746 - 2 | *M.tb* Rv1746 delete 2 bp | This study |
| ∆Rv1753c - 1 | *M.tb* Rv1753c delete 1 bp | This study |
| ∆Rv1753c - 2 | *M.tb* Rv1753c insert 1 bp | This study |
| ∆Rv1754c - 1 | *M.tb* Rv1754c delete 5 bp | This study |
| ∆Rv1754c - 2 | *M.tb* Rv1754c delete 10 bp | This study |
| ∆Rv1758 - 1 | *M.tb* Rv1758 delete 13 bp | This study |
| ∆Rv1758 - 2 | *M.tb* Rv1758 delete 4 bp | This study |
| ∆Rv1771 - 1 | *M.tb* Rv1771 delete 14 bp | This study |
| ∆Rv1771 - 2 | *M.tb* Rv1771 delete 5 bp | This study |
| ∆Rv1789 - 1 | *M.tb* Rv1789 delete 7 bp | This study |
| ∆Rv1789 - 2 | *M.tb* Rv1789 insert 1 bp | This study |
| ∆Rv1807 - 1 | *M.tb* Rv1807 delete 2 bp | This study |
| ∆Rv1807 - 2 | *M.tb* Rv1807 insert 1 bp | This study |
| ∆Rv1812c - 1 | *M.tb* Rv1812c delete 4 bp | This study |
| ∆Rv1812c - 2 | *M.tb* Rv1812c delete 2 bp | This study |
| ∆Rv1819c - 1 | *M.tb* Rv1819c insert 1 bp | This study |
| ∆Rv1819c - 2 | *M.tb* Rv1819c insert 217 bp | This study |
| ∆Rv1869c - 1 | *M.tb* Rv1869c insert 2 bp | This study |
| ∆Rv1869c - 2 | *M.tb* Rv1869c insert 1 bp | This study |
| ∆Rv1911c - 1 | *M.tb* Rv1911c delete 14 bp | This study |
| ∆Rv1911c - 2 | *M.tb* Rv1911c insert 1 bp | This study |
| ∆Rv1917c - 1 | *M.tb* Rv1917c insert 1 bp | This study |
| ∆Rv1917c - 2 | *M.tb* Rv1917c insert 1 bp | This study |
| ∆Rv1938 - 1 | *M.tb* Rv1938 delete 1 bp | This study |
| ∆Rv1938 - 2 | *M.tb* Rv1938 insert 1 bp | This study |
| ∆Rv1966 - 1 | *M.tb* Rv1966 delete 14 bp | This study |
| ∆Rv1966 - 2 | *M.tb* Rv1966 insert 1 bp | This study |
| ∆Rv1968 - 1 | *M.tb* Rv1968 delete 8 bp | This study |
| ∆Rv1968 - 2 | *M.tb* Rv1968 insert 1 bp | This study |
| ∆Rv1984c - 1 | *M.tb* Rv1984c delete 14 bp | This study |
| ∆Rv1984c - 2 | *M.tb* Rv1984c delete 5 bp | This study |
| ∆Rv1997 - 1 | *M.tb* Rv1997 insert 1 bp | This study |
| ∆Rv1997 - 2 | *M.tb* Rv1997 insert 1 bp | This study |
| ∆Rv2006 - 1 | *M.tb* Rv2006 delete 17 bp | This study |
| ∆Rv2006 - 2 | *M.tb* Rv2006 insert 439 bp | This study |
| ∆Rv2030c - 1 | *M.tb* Rv2030c delete 4 bp | This study |
| ∆Rv2030c - 2 | *M.tb* Rv2030c insert 1 bp | This study |
| ∆Rv2072c - 1 | *M.tb* Rv2072c insert 1 bp | This study |
| ∆Rv2072c - 2 | *M.tb* Rv2072c insert 1 bp | This study |
| ∆Rv2088 - 1 | *M.tb* Rv2088 delete 11 bp | This study |
| ∆Rv2088 - 2 | *M.tb* Rv2088 delete 2 bp | This study |
| ∆Rv2092c - 1 | *M.tb* Rv2092c delete 5 bp | This study |
| ∆Rv2092c - 2 | *M.tb* Rv2092c insert 2 bp | This study |
| ∆Rv2114 - 1 | *M.tb* Rv2114 delete 7 bp | This study |
| ∆Rv2114 - 2 | *M.tb* Rv2114 delete 8 bp | This study |
| ∆Rv2227 - 1 | *M.tb* Rv2227 delete 13 bp | This study |
| ∆Rv2227 - 2 | *M.tb* Rv2227 delete 10 bp | This study |
| ∆Rv2234 - 1 | *M.tb* Rv2234 delete 11 bp | This study |
| ∆Rv2234 - 2 | *M.tb* Rv2234 delete 10 bp | This study |
| ∆Rv2258c - 1 | *M.tb* Rv2258c delete 13 bp | This study |
| ∆Rv2258c - 2 | *M.tb* Rv2258c insert 1 bp | This study |
| ∆Rv2260 - 1 | *M.tb* Rv2260 delete 643 bp | This study |
| ∆Rv2260 - 2 | *M.tb* Rv2260 insert 1 bp | This study |
| ∆Rv2284 - 1 | *M.tb* Rv2284 delete 11 bp | This study |
| ∆Rv2284 - 2 | *M.tb* Rv2284 insert 31 bp | This study |
| ∆Rv2295 - 1 | *M.tb* Rv2295 delete 11 bp | This study |
| ∆Rv2295 - 2 | *M.tb* Rv2295 delete 10 bp | This study |
| ∆Rv2296 - 1 | *M.tb* Rv2296 insert 1 bp | This study |
| ∆Rv2296 - 2 | *M.tb* Rv2296 insert 50 bp | This study |
| ∆Rv2320c - 1 | *M.tb* Rv2320c insert 1 bp | This study |
| ∆Rv2320c - 2 | *M.tb* Rv2320c insert 1 bp | This study |
| ∆Rv2340c - 1 | *M.tb* Rv2340c delete 8 bp | This study |
| ∆Rv2340c - 2 | *M.tb* Rv2340c insert 1 bp | This study |
| ∆Rv2430c - 1 | *M.tb* Rv2430c delete 13 bp | This study |
| ∆Rv2430c - 2 | *M.tb* Rv2430c delete 7 bp | This study |
| ∆Rv2521 - 1 | *M.tb* Rv2521 delete 4 bp | This study |
| ∆Rv2521 - 2 | *M.tb* Rv2521 delete 11 bp | This study |
| ∆Rv2577 - 1 | *M.tb* Rv2577 delete 8 bp | This study |
| ∆Rv2577 - 2 | *M.tb* Rv2577 insert 1 bp | This study |
| ∆Rv2601 - 1 | *M.tb* Rv2601 delete 7 bp | This study |
| ∆Rv2601 - 2 | *M.tb* Rv2601 insert 112 bp | This study |
| ∆Rv2641 - 1 | *M.tb* Rv2641 insert 1 bp | This study |
| ∆Rv2641 - 2 | *M.tb* Rv2641 insert 1 bp | This study |
| ∆Rv2765 - 1 | *M.tb* Rv2765 delete 13 bp | This study |
| ∆Rv2765 - 2 | *M.tb* Rv2765 insert 1 bp | This study |
| ∆Rv2790c - 1 | *M.tb* Rv2790c insert 464 bp | This study |
| ∆Rv2790c - 2 | *M.tb* Rv2790c insert 1 bp | This study |
| ∆Rv2812 - 1 | *M.tb* Rv2812 insert 1 bp | This study |
| ∆Rv2812 - 2 | *M.tb* Rv2812 insert 1 bp | This study |
| ∆Rv2855 - 1 | *M.tb* Rv2855 insert 1 bp | This study |
| ∆Rv2855 - 2 | *M.tb* Rv2855 insert 1 bp | This study |
| ∆Rv3060c - 1 | *M.tb* Rv3060c insert 1 bp | This study |
| ∆Rv3060c - 2 | *M.tb* Rv3060c insert 1 bp | This study |
| ∆Rv3061c - 1 | *M.tb* Rv3061c delete 8 bp | This study |
| ∆Rv3061c - 2 | *M.tb* Rv3061c delete 2 bp | This study |
| ∆Rv3080c - 1 | *M.tb* Rv3080c delete 13 bp | This study |
| ∆Rv3080c - 2 | *M.tb* Rv3080c insert 196 bp | This study |
| ∆Rv3139 - 1 | *M.tb* Rv3139 delete 17 bp | This study |
| ∆Rv3139 - 2 | *M.tb* Rv3139 insert 1 bp | This study |
| ∆Rv3153 - 1 | *M.tb* Rv3153 delete 16 bp | This study |
| ∆Rv3153 - 2 | *M.tb* Rv3153 insert 2 bp | This study |
| ∆Rv3253c - 1 | *M.tb* Rv3253c delete 13 bp | This study |
| ∆Rv3253c - 2 | *M.tb* Rv3253c insert 1 bp | This study |
| ∆Rv3283 - 1 | *M.tb* Rv3283 delete 2 bp | This study |
| ∆Rv3283 - 2 | *M.tb* Rv3283 insert 1 bp | This study |
| ∆Rv3304 - 1 | *M.tb* Rv3304 delete 1 bp | This study |
| ∆Rv3304 - 2 | *M.tb* Rv3304 insert 41 bp | This study |
| ∆Rv3310 - 1 | *M.tb* Rv3310 delete 11 bp | This study |
| ∆Rv3310 - 2 | *M.tb* Rv3310 insert 1 bp | This study |
| ∆Rv3438 - 1 | *M.tb* Rv3438 delete 4 bp | This study |
| ∆Rv3438 - 2 | *M.tb* Rv3438 insert 1 bp | This study |
| ∆Rv3476c - 1 | *M.tb* Rv3476c delete 16 bp | This study |
| ∆Rv3476c - 2 | *M.tb* Rv3476c delete 5 bp | This study |
| ∆Rv3524 - 1 | *M.tb* Rv3524 delete 4 bp | This study |
| ∆Rv3524 - 2 | *M.tb* Rv3524 insert 2 bp | This study |
| ∆Rv3537 - 1 | *M.tb* Rv3537 delete 11 bp | This study |
| ∆Rv3537 - 2 | *M.tb* Rv3537 insert 1 bp | This study |
| ∆Rv3548c - 1 | *M.tb* Rv3548c delete 4 bp | This study |
| ∆Rv3548c - 2 | *M.tb* Rv3548c insert 1 bp | This study |
| ∆Rv3569c - 1 | *M.tb* Rv3569c delete 11 bp | This study |
| ∆Rv3569c - 2 | *M.tb* Rv3569c insert 1 bp | This study |
| ∆Rv3707c - 1 | *M.tb* Rv3707c insert 1 bp | This study |
| ∆Rv3707c - 2 | *M.tb* Rv3707c insert 47 bp | This study |
| ∆Rv3725 - 1 | *M.tb* Rv3725 insert 1 bp | This study |
| ∆Rv3725 - 2 | *M.tb* Rv3725 insert 2 bp | This study |
| ∆Rv3730c - 1 | *M.tb* Rv3730c delete 7 bp | This study |
| ∆Rv3730c - 2 | *M.tb* Rv3730c insert 1 bp | This study |
| ∆Rv3774 - 1 | *M.tb* Rv3774 delete 5 bp | This study |
| ∆Rv3774 - 2 | *M.tb* Rv3774 delete 13 bp | This study |
| ∆Rv3786c - 1 | *M.tb* Rv3786c delete 1 bp | This study |
| ∆Rv3786c - 2 | *M.tb* Rv3786c insert 1 bp | This study |
| ∆Rv3811 - 1 | *M.tb* Rv3811 delete 7 bp | This study |
| ∆Rv3811 - 2 | *M.tb* Rv3811 insert 157 bp | This study |
| ∆Rv3852 - 1 | *M.tb* Rv3852 insert 164 bp | This study |
| ∆Rv3852 - 2 | *M.tb* Rv3852 insert 29 bp | This study |
| ∆Rv3873 - 1 | *M.tb* Rv3873 insert 1 bp | This study |
| ∆Rv3873 - 2 | *M.tb* Rv3873 insert 1 bp | This study |
| WT - sg_80042 | The WT was transferred to the control plasmid | This study |
| WT - sg_80043 | The WT was transferred to the control plasmid | This study |
| WT - sg_80044 | The WT was transferred to the control plasmid | This study |

**Table S4. Plasmids**

| **Name** | **Description** | **Source** |
| --- | --- | --- |
| pYC1446 | The sgRNA integrated plasmid was used for CRISPR library construction | Y. Sun, *et al* |
| pYC1759 | pNHEJ-recX-sacB，with the kanamycin resistance element, the helper plasmid | Y. Sun, *et al* |
| pYC1876 | sgRNA plasmids，for construct the mutants | Y. Sun, *et al* |
| pYC1876-Rv0066c | sgRNA plasmids，for construct the mutants M.tb ΔRv0066c | This study |
| pYC1876-Rv3139 | sgRNA plasmids，for construct the mutants M.tb ΔRv3139 | This study |
| pYC1446 - Rv0062 | The sgRNA integrated plasmid was used for M.tb mutant | This study |
| pYC1446 - Rv0066c | The sgRNA integrated plasmid was used for M.tb mutant | This study |
| pYC1446 - Rv0089 | The sgRNA integrated plasmid was used for M.tb mutant | This study |
| pYC1446 - Rv0116c | The sgRNA integrated plasmid was used for M.tb mutant | This study |
| pYC1446 - Rv0139 | The sgRNA integrated plasmid was used for M.tb mutant | This study |
| pYC1446 - Rv0153c | The sgRNA integrated plasmid was used for M.tb mutant | This study |
| pYC1446 - Rv0169 | The sgRNA integrated plasmid was used for M.tb mutant | This study |
| pYC1446 - Rv0183 | The sgRNA integrated plasmid was used for M.tb mutant | This study |
| pYC1446 - Rv0187 | The sgRNA integrated plasmid was used for M.tb mutant | This study |
| pYC1446 - Rv0198c | The sgRNA integrated plasmid was used for M.tb mutant | This study |
| pYC1446 - Rv0229c | The sgRNA integrated plasmid was used for M.tb mutant | This study |
| pYC1446 - Rv0260c | The sgRNA integrated plasmid was used for M.tb mutant | This study |
| pYC1446 - Rv0274 | The sgRNA integrated plasmid was used for M.tb mutant | This study |
| pYC1446 - Rv0305c | The sgRNA integrated plasmid was used for M.tb mutant | This study |
| pYC1446 - Rv0386 | The sgRNA integrated plasmid was used for M.tb mutant | This study |
| pYC1446 - Rv0410c | The sgRNA integrated plasmid was used for M.tb mutant | This study |
| pYC1446 - Rv0411c | The sgRNA integrated plasmid was used for M.tb mutant | This study |
| pYC1446 - Rv0425c | The sgRNA integrated plasmid was used for M.tb mutant | This study |
| pYC1446 - Rv0426c | The sgRNA integrated plasmid was used for M.tb mutant | This study |
| pYC1446 - Rv0432 | The sgRNA integrated plasmid was used for M.tb mutant | This study |
| pYC1446 - Rv0435c | The sgRNA integrated plasmid was used for M.tb mutant | This study |
| pYC1446 - Rv0442c | The sgRNA integrated plasmid was used for M.tb mutant | This study |
| pYC1446 - Rv0446c | The sgRNA integrated plasmid was used for M.tb mutant | This study |
| pYC1446 - Rv0457c | The sgRNA integrated plasmid was used for M.tb mutant | This study |
| pYC1446 - Rv0518 | The sgRNA integrated plasmid was used for M.tb mutant | This study |
| pYC1446 - Rv0561c | The sgRNA integrated plasmid was used for M.tb mutant | This study |
| pYC1446 - Rv0571c | The sgRNA integrated plasmid was used for M.tb mutant | This study |
| pYC1446 - Rv0577 | The sgRNA integrated plasmid was used for M.tb mutant | This study |
| pYC1446 - Rv0594 | The sgRNA integrated plasmid was used for M.tb mutant | This study |
| pYC1446 - Rv0618 | The sgRNA integrated plasmid was used for M.tb mutant | This study |
| pYC1446 - Rv0622 | The sgRNA integrated plasmid was used for M.tb mutant | This study |
| pYC1446 - Rv0629c | The sgRNA integrated plasmid was used for M.tb mutant | This study |
| pYC1446 - Rv0648 | The sgRNA integrated plasmid was used for M.tb mutant | This study |
| pYC1446 - Rv0654 | The sgRNA integrated plasmid was used for M.tb mutant | This study |
| pYC1446 - Rv0669c | The sgRNA integrated plasmid was used for M.tb mutant | This study |
| pYC1446 - Rv0671 | The sgRNA integrated plasmid was used for M.tb mutant | This study |
| pYC1446 - Rv0733 | The sgRNA integrated plasmid was used for M.tb mutant | This study |
| pYC1446 - Rv0755c | The sgRNA integrated plasmid was used for M.tb mutant | This study |
| pYC1446 - Rv0774c | The sgRNA integrated plasmid was used for M.tb mutant | This study |
| pYC1446 - Rv0794c | The sgRNA integrated plasmid was used for M.tb mutant | This study |
| pYC1446 - Rv0800 | The sgRNA integrated plasmid was used for M.tb mutant | This study |
| pYC1446 - Rv0806c | The sgRNA integrated plasmid was used for M.tb mutant | This study |
| pYC1446 - Rv0838 | The sgRNA integrated plasmid was used for M.tb mutant | This study |
| pYC1446 - Rv0861c | The sgRNA integrated plasmid was used for M.tb mutant | This study |
| pYC1446 - Rv0862c | The sgRNA integrated plasmid was used for M.tb mutant | This study |
| pYC1446 - Rv0878c | The sgRNA integrated plasmid was used for M.tb mutant | This study |
| pYC1446 - Rv0887c | The sgRNA integrated plasmid was used for M.tb mutant | This study |
| pYC1446 - Rv0907 | The sgRNA integrated plasmid was used for M.tb mutant | This study |
| pYC1446 - Rv0911 | The sgRNA integrated plasmid was used for M.tb mutant | This study |
| pYC1446 - Rv0913c | The sgRNA integrated plasmid was used for M.tb mutant | This study |
| pYC1446 - Rv0920c | The sgRNA integrated plasmid was used for M.tb mutant | This study |
| pYC1446 - Rv0922 | The sgRNA integrated plasmid was used for M.tb mutant | This study |
| pYC1446 - Rv0931c | The sgRNA integrated plasmid was used for M.tb mutant | This study |
| pYC1446 - Rv0938 | The sgRNA integrated plasmid was used for M.tb mutant | This study |
| pYC1446 - Rv0977 | The sgRNA integrated plasmid was used for M.tb mutant | This study |
| pYC1446 - Rv0980c | The sgRNA integrated plasmid was used for M.tb mutant | This study |
| pYC1446 - Rv0988 | The sgRNA integrated plasmid was used for M.tb mutant | This study |
| pYC1446 - Rv1050 | The sgRNA integrated plasmid was used for M.tb mutant | This study |
| pYC1446 - Rv1057 | The sgRNA integrated plasmid was used for M.tb mutant | This study |
| pYC1446 - Rv1073 | The sgRNA integrated plasmid was used for M.tb mutant | This study |
| pYC1446 - Rv1079 | The sgRNA integrated plasmid was used for M.tb mutant | This study |
| pYC1446 - Rv1084 | The sgRNA integrated plasmid was used for M.tb mutant | This study |
| pYC1446 - Rv1090 | The sgRNA integrated plasmid was used for M.tb mutant | This study |
| pYC1446 - Rv1124 | The sgRNA integrated plasmid was used for M.tb mutant | This study |
| pYC1446 - Rv1135c | The sgRNA integrated plasmid was used for M.tb mutant | This study |
| pYC1446 - Rv1162 | The sgRNA integrated plasmid was used for M.tb mutant | This study |
| pYC1446 - Rv1243c | The sgRNA integrated plasmid was used for M.tb mutant | This study |
| pYC1446 - Rv1259 | The sgRNA integrated plasmid was used for M.tb mutant | This study |
| pYC1446 - Rv1266c | The sgRNA integrated plasmid was used for M.tb mutant | This study |
| pYC1446 - Rv1268c | The sgRNA integrated plasmid was used for M.tb mutant | This study |
| pYC1446 - Rv1317c | The sgRNA integrated plasmid was used for M.tb mutant | This study |
| pYC1446 - Rv1377c | The sgRNA integrated plasmid was used for M.tb mutant | This study |
| pYC1446 - Rv1386 | The sgRNA integrated plasmid was used for M.tb mutant | This study |
| pYC1446 - Rv1407 | The sgRNA integrated plasmid was used for M.tb mutant | This study |
| pYC1446 - Rv1426c | The sgRNA integrated plasmid was used for M.tb mutant | This study |
| pYC1446 - Rv1454c | The sgRNA integrated plasmid was used for M.tb mutant | This study |
| pYC1446 - Rv1468c | The sgRNA integrated plasmid was used for M.tb mutant | This study |
| pYC1446 - Rv1515c | The sgRNA integrated plasmid was used for M.tb mutant | This study |
| pYC1446 - Rv1548c | The sgRNA integrated plasmid was used for M.tb mutant | This study |
| pYC1446 - Rv1635c | The sgRNA integrated plasmid was used for M.tb mutant | This study |
| pYC1446 - Rv1665 | The sgRNA integrated plasmid was used for M.tb mutant | This study |
| pYC1446 - Rv1677 | The sgRNA integrated plasmid was used for M.tb mutant | This study |
| pYC1446 - Rv1679 | The sgRNA integrated plasmid was used for M.tb mutant | This study |
| pYC1446 - Rv1688 | The sgRNA integrated plasmid was used for M.tb mutant | This study |
| pYC1446 - Rv1743 | The sgRNA integrated plasmid was used for M.tb mutant | This study |
| pYC1446 - Rv1746 | The sgRNA integrated plasmid was used for M.tb mutant | This study |
| pYC1446 - Rv1753c | The sgRNA integrated plasmid was used for M.tb mutant | This study |
| pYC1446 - Rv1754c | The sgRNA integrated plasmid was used for M.tb mutant | This study |
| pYC1446 - Rv1758 | The sgRNA integrated plasmid was used for M.tb mutant | This study |
| pYC1446 - Rv1771 | The sgRNA integrated plasmid was used for M.tb mutant | This study |
| pYC1446 - Rv1789 | The sgRNA integrated plasmid was used for M.tb mutant | This study |
| pYC1446 - Rv1807 | The sgRNA integrated plasmid was used for M.tb mutant | This study |
| pYC1446 - Rv1812c | The sgRNA integrated plasmid was used for M.tb mutant | This study |
| pYC1446 - Rv1819c | The sgRNA integrated plasmid was used for M.tb mutant | This study |
| pYC1446 - Rv1869c | The sgRNA integrated plasmid was used for M.tb mutant | This study |
| pYC1446 - Rv1911c | The sgRNA integrated plasmid was used for M.tb mutant | This study |
| pYC1446 - Rv1917c | The sgRNA integrated plasmid was used for M.tb mutant | This study |
| pYC1446 - Rv1918c | The sgRNA integrated plasmid was used for M.tb mutant | This study |
| pYC1446 - Rv1938 | The sgRNA integrated plasmid was used for M.tb mutant | This study |
| pYC1446 - Rv1966 | The sgRNA integrated plasmid was used for M.tb mutant | This study |
| pYC1446 - Rv1968 | The sgRNA integrated plasmid was used for M.tb mutant | This study |
| pYC1446 - Rv1984c | The sgRNA integrated plasmid was used for M.tb mutant | This study |
| pYC1446 - Rv1997 | The sgRNA integrated plasmid was used for M.tb mutant | This study |
| pYC1446 - Rv2006 | The sgRNA integrated plasmid was used for M.tb mutant | This study |
| pYC1446 - Rv2030c | The sgRNA integrated plasmid was used for M.tb mutant | This study |
| pYC1446 - Rv2072c | The sgRNA integrated plasmid was used for M.tb mutant | This study |
| pYC1446 - Rv2088 | The sgRNA integrated plasmid was used for M.tb mutant | This study |
| pYC1446 - Rv2092c | The sgRNA integrated plasmid was used for M.tb mutant | This study |
| pYC1446 - Rv2114 | The sgRNA integrated plasmid was used for M.tb mutant | This study |
| pYC1446 - Rv2227 | The sgRNA integrated plasmid was used for M.tb mutant | This study |
| pYC1446 - Rv2234 | The sgRNA integrated plasmid was used for M.tb mutant | This study |
| pYC1446 - Rv2258c | The sgRNA integrated plasmid was used for M.tb mutant | This study |
| pYC1446 - Rv2260 | The sgRNA integrated plasmid was used for M.tb mutant | This study |
| pYC1446 - Rv2284 | The sgRNA integrated plasmid was used for M.tb mutant | This study |
| pYC1446 - Rv2295 | The sgRNA integrated plasmid was used for M.tb mutant | This study |
| pYC1446 - Rv2296 | The sgRNA integrated plasmid was used for M.tb mutant | This study |
| pYC1446 - Rv2320c | The sgRNA integrated plasmid was used for M.tb mutant | This study |
| pYC1446 - Rv2340c | The sgRNA integrated plasmid was used for M.tb mutant | This study |
| pYC1446 - Rv2430c | The sgRNA integrated plasmid was used for M.tb mutant | This study |
| pYC1446 - Rv2521 | The sgRNA integrated plasmid was used for M.tb mutant | This study |
| pYC1446 - Rv2577 | The sgRNA integrated plasmid was used for M.tb mutant | This study |
| pYC1446 - Rv2601 | The sgRNA integrated plasmid was used for M.tb mutant | This study |
| pYC1446 - Rv2641 | The sgRNA integrated plasmid was used for M.tb mutant | This study |
| pYC1446 - Rv2765 | The sgRNA integrated plasmid was used for M.tb mutant | This study |
| pYC1446 - Rv2790c | The sgRNA integrated plasmid was used for M.tb mutant | This study |
| pYC1446 - Rv2793c | The sgRNA integrated plasmid was used for M.tb mutant | This study |
| pYC1446 - Rv2812 | The sgRNA integrated plasmid was used for M.tb mutant | This study |
| pYC1446 - Rv2854 | The sgRNA integrated plasmid was used for M.tb mutant | This study |
| pYC1446 - Rv2855 | The sgRNA integrated plasmid was used for M.tb mutant | This study |
| pYC1446 - Rv2914c | The sgRNA integrated plasmid was used for M.tb mutant | This study |
| pYC1446 - Rv3060c | The sgRNA integrated plasmid was used for M.tb mutant | This study |
| pYC1446 - Rv3061c | The sgRNA integrated plasmid was used for M.tb mutant | This study |
| pYC1446 - Rv3080c | The sgRNA integrated plasmid was used for M.tb mutant | This study |
| pYC1446 - Rv3139 | The sgRNA integrated plasmid was used for M.tb mutant | This study |
| pYC1446 - Rv3153 | The sgRNA integrated plasmid was used for M.tb mutant | This study |
| pYC1446 - Rv3159c | The sgRNA integrated plasmid was used for M.tb mutant | This study |
| pYC1446 - Rv3253c | The sgRNA integrated plasmid was used for M.tb mutant | This study |
| pYC1446 - Rv3274c | The sgRNA integrated plasmid was used for M.tb mutant | This study |
| pYC1446 - Rv3283 | The sgRNA integrated plasmid was used for M.tb mutant | This study |
| pYC1446 - Rv3304 | The sgRNA integrated plasmid was used for M.tb mutant | This study |
| pYC1446 - Rv3310 | The sgRNA integrated plasmid was used for M.tb mutant | This study |
| pYC1446 - Rv3438 | The sgRNA integrated plasmid was used for M.tb mutant | This study |
| pYC1446 - Rv3476c | The sgRNA integrated plasmid was used for M.tb mutant | This study |
| pYC1446 - Rv3524 | The sgRNA integrated plasmid was used for M.tb mutant | This study |
| pYC1446 - Rv3533c | The sgRNA integrated plasmid was used for M.tb mutant | This study |
| pYC1446 - Rv3537 | The sgRNA integrated plasmid was used for M.tb mutant | This study |
| pYC1446 - Rv3548c | The sgRNA integrated plasmid was used for M.tb mutant | This study |
| pYC1446 - Rv3558 | The sgRNA integrated plasmid was used for M.tb mutant | This study |
| pYC1446 - Rv3569c | The sgRNA integrated plasmid was used for M.tb mutant | This study |
| pYC1446 - Rv3699 | The sgRNA integrated plasmid was used for M.tb mutant | This study |
| pYC1446 - Rv3707c | The sgRNA integrated plasmid was used for M.tb mutant | This study |
| pYC1446 - Rv3724B | The sgRNA integrated plasmid was used for M.tb mutant | This study |
| pYC1446 - Rv3725 | The sgRNA integrated plasmid was used for M.tb mutant | This study |
| pYC1446 - Rv3730c | The sgRNA integrated plasmid was used for M.tb mutant | This study |
| pYC1446 - Rv3774 | The sgRNA integrated plasmid was used for M.tb mutant | This study |
| pYC1446 - Rv3786c | The sgRNA integrated plasmid was used for M.tb mutant | This study |
| pYC1446 - Rv3811 | The sgRNA integrated plasmid was used for M.tb mutant | This study |
| pYC1446 - Rv3852 | The sgRNA integrated plasmid was used for M.tb mutant | This study |
| pYC1446 - Rv3873 | The sgRNA integrated plasmid was used for M.tb mutant | This study |

**Table S5. All primers used for PCR validation of the mutant strains**

| **Number** | **Genes** | **Forward Primer (5’ to 3’)** | **Reverse Primer (5’ to 3’)** |
| --- | --- | --- | --- |
| 1 | Rv0062 | gttgcagccgttgcttccat | atccacccgcggtaatcagt |
| 2 | Rv0066c | cgctacaacgcggagtgatt | cagcgactcgatcttgctgt |
| 3 | Rv0089 | catccactacgacgctctgc | aagtgccataagccgtttcg |
| 4 | Rv0116c | gcaatgggtcgatggtcact | gaagtaccatgccgcgttgt |
| 5 | Rv0139 | catctacaacccgttgcggt | accgtcgcataactgctggt |
| 6 | Rv0153c | gatgtcatcgaccgcgcaat | tcgcttacgcgaatcggatt |
| 7 | Rv0169 | aggtcgttgtcctgtttgca | gaagaacagcagcaccaccg |
| 8 | Rv0183 | tgcgagcacactcacgtcaa | gccaaaactagtggagggct |
| 9 | Rv0187 | ggtggtcagctgtcggtaat | gctccaagattacgcccgtt |
| 10 | Rv0198c | ctgagccacatcgacgctga | gtcgggtaatccgatgccgg |
| 11 | Rv0229c | acaccatgctcgtcgacaac | cgaagtccgcgtcgtaatgt |
| 12 | Rv0260c | tggtaacgcgactgaaacct | accactgttgccacgatgtt |
| 13 | Rv0274 | cgcctctttggagtcgtagt | gccagatggttcatggtgct |
| 14 | Rv0305c | agcctgaacgtacctccgac | accgatcaggccttggtagt |
| 15 | Rv0386 | tggctagcatgagtgcgact | cgacaagttgccagcaacct |
| 16 | Rv0410c | acgttgtatcgcaagtggct | caattgcgcggacatctctt |
| 17 | Rv0411c | caggtggcactgggtaaagg | cgaggtagacggtggagaag |
| 18 | Rv0425c | gcgttgctattgcggaagct | cagtgatgtgtcgggatcgt |
| 19 | Rv0426c | cagttgcgcgttgtctcagt | cgtgcgatggatgcagcaat |
| 20 | Rv0432 | gtcctgtccgcgttgtttct | tgacctggacgtagcgttct |
| 21 | Rv0435c | ccaatgccattgctgccctt | gtgctgaggtggcgatcaat |
| 22 | Rv0442c | acggtcgctgacagttaagt | gcgttccagaagctggtgtt |
| 23 | Rv0446c | cgaggtgtttgcacggatgt | gagtcaaactccgacgcgtt |
| 24 | Rv0457c | gttctcgaagtagcgcccat | ctccgaaccacgcaattcgt |
| 25 | Rv0518 | gacgcgagttcacatcggtt | gctaaaccctttgcgtggct |
| 26 | Rv0561c | cgaatcgctcatcacctcgt | ccgatgttcacttcgccgtt |
| 27 | Rv0571c | ccatgagatggcgacacact | gaccacggtgagttggcatt |
| 28 | Rv0577 | tcgaccttcagaccaccgat | cttgtccgtgagcagttcgt |
| 29 | Rv0594 | gcatctcgggaatgttgcgt | gccggaattgacctggctat |
| 30 | Rv0618 | gacctgcccgatgacacaag | gcgaaaagaacagcaggtca |
| 31 | Rv0622 | cgtgcggcgcatacaagatt | gttcgcgtcggagttgaact |
| 32 | Rv0629c | tcggtgtgtgtggatctgtt | gtcgaccacgatcacgttgt |
| 33 | Rv0648 | ccattacatgccggcgcatt | gaggtagacctggtcggact |
| 34 | Rv0755c | acaaatgcgaactcgcaggt | cccaggttgaagttgccgat |
| 35 | Rv0654 | agggcgtaccgttccaatat | ggtcgcgatcaaacatccgt |
| 36 | Rv0669c | caccagcgacttaggtcacg | ccgcgaaacccttgttgtct |
| 37 | Rv0671 | aggacgacatcgcgtttctg | gcgccagaacgcattgagat |
| 38 | Rv0733 | tgtgctgagccggattacct | tcgagactcaggctggatgt |
| 39 | Rv0774c | atgctgccagtcaaagtggt | acgtgcacacgacgtttgtc |
| 40 | Rv0794c | tgagccagacaccaggtgat | tgtctccaagccgatgtcgt |
| 41 | Rv0800 | cctgcaataccggtgatgct | gcatagcaactggcctggtt |
| 42 | Rv0806c | aggtttccggccgatgttct | ctccaccgcatgcgagttat |
| 43 | Rv0838 | gtcgatgttcgcggtgttgt | cactcaccggagtacaccgt |
| 44 | Rv0861c | ccatccaccgcatcacgttg | gagtaactgaccagcgcgtc |
| 45 | Rv0862c | tggtccacgtacctggtctt | tcgtcgattcccgttgcctt |
| 46 | Rv0878c | gcctcgttttcgttgctgat | atgcccaagaagttccccac |
| 47 | Rv0887c | ctgtcaccacaccttgtcgt | cgcaacaagttcaccgacgt |
| 48 | Rv0907 | gcgatcaaaccaccgagtgt | aggtcacgacgttgtcggtt |
| 49 | Rv0911 | gctggatcgacttgacgact | aattcgacggaacgccttcg |
| 50 | Rv0913c | ccaccacggtcagtttcgtt | cacgaacgttggatcggctt |
| 51 | Rv0920c | cactcaagggcatggatgct | tcgcggatcttgaccatgat |
| 52 | Rv0922 | tttcggtgctgttcgagctt | cgctgcgacaaggtcatgtt |
| 53 | Rv0931c | cacacatcgtgccgatccat | gttgctcggatgtggtgagt |
| 54 | Rv0938 | gttacgctcgcgacttgtgt | tgacgaacgtattgccgtct |
| 55 | Rv0977 | cgcgggactatcaaacgctg | taccgccatcgccgttgatt |
| 56 | Rv0980c | ccgtggtatgcgggtttgtt | tccacccacaccgattaacc |
| 57 | Rv0988 | aagcaggattgaccggtgtt | gttgttggattcacgcgcat |
| 58 | Rv1050 | acctcacgatgagcgagctt | accaccgacgacatgttcat |
| 59 | Rv1057 | tcgctcgagagagcagagat | gatcgtgccgatgacatcgt |
| 60 | Rv1073 | cacgcattggtggagttgat | tctcggctgagacggtgttg |
| 61 | Rv1079 | acgttggcggacaagatcat | gatcagcgaggaattccgct |
| 62 | Rv1084 | tgccactggtgtcacgtcat | cagcgacgaggtgaacatgt |
| 63 | Rv1090 | tcaccgagatgaacggtgct | aggtaccacgagtcggtgat |
| 64 | Rv1124 | ccaccgtgcaacatcaggat | accgacatgatcaccgcctt |
| 65 | Rv1135c | gttgcctcccgaggtcaatt | attgccgttgcctaggttgt |
| 66 | Rv1162 | gcgttcgcgttcaactacct | cacacgtttccgagcacacc |
| 67 | Rv1243c | acacgcaatgcaccgtcaag | ctgcccaaggttggttccat |
| 68 | Rv1259 | atcagcgttgcgaacaggat | gacgatcgcacggatgtgat |
| 69 | Rv1266c | cattgttgcctggcaaggtt | ggtgcctaactgggtcagtt |
| 70 | Rv1268c | aaggtcaccatcggaacctt | atcatctcggcgttgatgct |
| 71 | Rv1317c | ggtgggcgggtgtaattgtt | gcgaacggtgtcgttgaact |
| 72 | Rv1377c | gccaggtggaagccagaatc | cagtggaacatcccgcagtc |
| 73 | Rv1386 | tcgtcacgctgtgttaacct | ggcataactggcacccgatt |
| 74 | Rv1407 | cgagctgacatacggcacct | taactggctgccctcgtcct |
| 75 | Rv1426c | tacaaggtaccgcgcgacat | acctctgctacgcatcggtt |
| 76 | Rv1454c | catcgacacctacttccgct | gacaccgtcgtaaacggctt |
| 77 | Rv1468c | caatgggtggggaatggtct | tgcacgaactggctgtgaaa |
| 78 | Rv1515c | tcatggccgactggatcctt | accgatcggtcaattgtcgt |
| 79 | Rv1548c | ctcattttcggcggtgacct | cacgctggcacttgatcagt |
| 80 | Rv1635c | gtggcgatgacacgacgatt | tggctgaagccgagatcgtt |
| 81 | Rv1665 | cagcgacgaggttatcgact | gcgatcatgtcgatgtcgct |
| 82 | Rv1677 | attggcgcacttaccgtagt | gtcagttcgtcctgcgacat |
| 83 | Rv1679 | gatcggacttgtcgcggtat | agccatgtccgccaacagat |
| 84 | Rv1688 | gatgaacgctgaggaactgg | ggactggacggatcaaacaa |
| 85 | Rv1743 | tcgcggttgctgaaaccatt | tgatccggtcaagcactcat |
| 86 | Rv1746 | ccacgattctcgtcgagtgt | accgcagtgatgatctcggt |
| 87 | Rv1753c | tggccatttgtgcgcgatat | ttggctatgcccaggttgat |
| 88 | Rv1754c | gatcggatcgtcgatgttcg | ttcggagttagctgagtggc |
| 89 | Rv1758 | acctcctcgatgaaccacct | tatagcggaccgatgacgat |
| 90 | Rv1771 | acttcgacaccatcggctca | cagattctggaaacgcaccc |
| 91 | Rv1789 | gcggcgattcacgagatgtt | agggtcttggccgactgaat |
| 92 | Rv1807 | gccatgaatgaggcgttcgt | aagggcgcaatcgtgttact |
| 93 | Rv1812c | tggcacgtgtggatgttcct | gtccttgcagatgctgctgt |
| 94 | Rv1819c | aacgtgaagaccgtgtcgat | gcgctgcgtcaagtagatgt |
| 95 | Rv1869c | agctcaaccacgttcgtcat | gtcggctgcagtgatctctt |
| 96 | Rv1911c | cgagagacaccgccatacgt | gaatgccggactgctgattg |
| 97 | Rv1917c | tggccctattcgggtgatat | tgcccgaagaagtttgagaa |
| 98 | Rv1918c | gcgtgtgacctttcatcagc | tccagagcccacatctcctc |
| 99 | Rv1938 | tactcgtggcggcatcagat | gcggtagttcggcatgactt |
| 100 | Rv1966 | aactttcacctggcgaccta | ctggcctatccgttcacctt |
| 101 | Rv1968 | tcttcgccttctacctgtgc | tggttttgattgccacttcg |
| 102 | Rv1984c | tccggtgcatattggatcgt | gcatcgttggtgtcgtggtt |
| 103 | Rv1997 | gacttacggccctgatccac | gcattgtgtgctcgtgaccc |
| 104 | Rv2006 | taccacgacgctgtcatcgt | gcgtcgggaatcgtggagat |
| 105 | Rv2030c | gtgctgacgaggacgacatt | aatcacgatctgcgctggtt |
| 106 | Rv2072c | ggatggggctgacctacacg | cgccgagatagtgctggaat |
| 107 | Rv2088 | agttggctatcgtggtttgg | aaatcgcttagcagcactcg |
| 108 | Rv2092c | tgacaagcgaaaagccaagc | gaggaaatgcacctcatcca |
| 109 | Rv2114 | cttcgggaatggacgcacta | acctggctgacggtgtatgg |
| 110 | Rv2227 | tcacctcggattgccgttag | gtgtagggcgttccagtcgt |
| 111 | Rv2234 | gccacttcggaattgagcag | tgacgttttggcattcttgc |
| 112 | Rv2258c | gcggtaggtcaggtcggtat | ggtattcgctgtagggcaca |
| 113 | Rv2260 | gaggaggcgttccacaagat | ggcatcgaatgtcacgctct |
| 114 | Rv2284 | tcgctgtggtcgtggtttat | gggctgtgtcggtagttgat |
| 115 | Rv2295 | acctcgggtgacggtcttgt | catcgacatagtgcggtgca |
| 116 | Rv2296 | gtttgcaccgcactatgtcg | aaccgaagatggcaaggaac |
| 117 | Rv2320c | gtcgtctggggagaatgtca | cccgtattccagcagtaggc |
| 118 | Rv2340c | cgacctcgacacttcagtgt | gatcttgggcgagttcgctg |
| 119 | Rv2430c | ggagtcggcatgtcttttgt | cctcatcacctctccgtcct |
| 120 | Rv2521 | aagttctccagtcgcccact | gcaccgtcttgccatacatc |
| 121 | Rv2577 | ggcgggtatgaaacgtgagt | ggttgaacaacggggcaata |
| 122 | Rv2601 | gtgagcacatcgacgcacct | ctcaatgtcgtgggaatgca |
| 123 | Rv2641 | caccctgccagataccttga | gtcacccacaccttgtcctg |
| 124 | Rv2765 | tcgtatggcgtcacgattga | agtagccgcagacaccgaag |
| 125 | Rv2790c | acgatgcctaaccaagggtc | tgtcgtccagggtgtattcg |
| 126 | Rv2793c | ccaagaggtggctgggtttc | tgtaggttcccgaggagcag |
| 127 | Rv2812 | gtcgatgccgatccggtgat | tcttgcggagatcaatccgt |
| 128 | Rv2854 | cggcaagaacggttcgtctg | gagcggttctgctcgttgat |
| 129 | Rv2855 | tcacacggtacgggaaaagg | gcggtacacgtcgatgttgg |
| 130 | Rv2914c | gtgaggtactcgccatcgtc | gtctgctttgagcgttggat |
| 131 | Rv3060c | cgcccttgactcagccaata | cctcaacgtgtcgttcgctt |
| 132 | Rv3061c | tgccgatcttagagagggtg | cgtcagggttccgtcaatca |
| 133 | Rv3080c | gtccttccctccgttggtct | tccaggtcggtgctcaatac |
| 134 | Rv3139 | ctatcagatgcctggcgtgt | cgggcgtcagatcgaagtag |
| 135 | Rv3153 | gttattgatccccgtctcgc | cgctcctcttcggtattgtc |
| 136 | Rv3159c | attcgttgcggatgtttacc | attcgtggcaccgtcatttc |
| 137 | Rv3253c | gcacaatctggacgttggag | ccgaaggtggcataagagaa |
| 138 | Rv3274c | cgcgactagcacggagatta | atcccgagcttgcgttcttt |
| 139 | Rv3283 | gtcgaatgtgatcggcttga | gtttggtcgtccgggtttat |
| 140 | Rv3304 | gggtcaccacagcccataac | agagcacgacgaacaccttc |
| 141 | Rv3310 | ctgatacgggtgcgtccatc | cagcgaacagtgccaggtag |
| 142 | Rv3438 | gctcgttggttggtggagtt | atgcgtagggtggccttatc |
| 143 | Rv3476c | acacggggtgaacgctacat | ggacgagccgacacaaaata |
| 144 | Rv3524 | gatccaagtcatgggcaagg | ggtccactgccaaaccatgc |
| 145 | Rv3533c | caccgtatgcgggatggttg | ggtgttaccgatgttgccgt |
| 146 | Rv3537 | cttcgctcggcttgtgctag | ggtgaaggcggtgttgagtt |
| 147 | Rv3548c | agtgacgagcaaaggagcaa | caccaagggtgagacgttct |
| 148 | Rv3558 | aggatcgaggggcttggtac | ggttggagctgcctttgttt |
| 149 | Rv3569c | ccaatgaggctccgattcaa | gaccccagatcagcaacacc |
| 150 | Rv3699 | caatggcacacgagggactg | cgacttcgtttggcttgact |
| 151 | Rv3707c | acaccatccagatcgccgag | gtagttgcggcggttgatct |
| 152 | Rv3724B | tcacgttgtcggattcactg | gaagcgttttcggctggtac |
| 153 | Rv3725 | ggactttgcggttgcagaca | tgacgctggtcatcctcctg |
| 154 | Rv3730c | atctccccgtcgatcacaca | cccgttcttccttccaccac |
| 155 | Rv3774 | gacccagcgatacgtctcct | cgcagtatttcggtgtggaa |
| 156 | Rv3786c | tctggggtgatagagaggcg | tgcgtagccaaacaacaaag |
| 157 | Rv3811 | ccgattccgtgtcgtctcat | ccagggtcttgctgtggtaa |
| 158 | Rv3852 | tcctggcatgatgggaccga | ctgcgagagcaccgacttca |
| 159 | Rv3873 | ctaaataccgcacggctgat | ggctgcctggttccacatac |
